# Guidance Receptor Degradation Is Required for Neuronal Connectivity in the *Drosophila* Nervous System

**DOI:** 10.1371/journal.pbio.1000553

**Published:** 2010-12-07

**Authors:** W. Ryan Williamson, Taehong Yang, Jonathan R. Terman, P. Robin Hiesinger

**Affiliations:** 1 Department of Physiology and Green Center for Systems Biology, University of Texas Southwestern Medical Center at Dallas, Dallas, Texas, United States of America; 2 Departments of Neuroscience and Pharmacology, University of Texas Southwestern Medical Center at Dallas, Dallas, Texas, United States of America; University of Cambridge, United Kingdom

## Abstract

Axon pathfinding and synapse formation rely on precise spatiotemporal localization of guidance receptors. However, little is known about the neuron-specific intracellular trafficking mechanisms that underlie the sorting and activity of these receptors. Here we show that loss of the neuron-specific v-ATPase subunit a1 leads to progressive endosomal guidance receptor accumulations after neuronal differentiation. In the embryo and in adult photoreceptors, these accumulations occur after axon pathfinding and synapse formation is complete. In contrast, receptor missorting occurs sufficiently early in neurons of the adult central nervous system to cause connectivity defects. An increase of guidance receptors, but not of membrane proteins without signaling function, causes specific gain-of-function phenotypes. A point mutant that promotes sorting but prevents degradation reveals spatiotemporally specific guidance receptor turnover and accelerates developmental defects in photoreceptors and embryonic motor neurons. Our findings indicate that a neuron-specific endolysosomal degradation mechanism is part of the cell biological machinery that regulates guidance receptor turnover and signaling.

## Abstract 2

During *Drosophila* brain development, a neuron-specific endolysosomal degradation pathway provides a mechanism for continuous guidance receptor turnover and proper connectivity.

## Author Summary

Brain wiring is determined by genetic and environmental factors, nature and nurture. The *Drosophila* brain is a model for the genetic basis of brain wiring. The fly visual system in particular is thought to be “hard-wired,” i.e., encoded solely by a genetic program. Some key genes encode the guidance receptors that serve as “wiring” and synaptic connectivity signals. However, it is poorly understood how guidance receptors are spatiotemporally regulated to serve as meaningful synapse formation signals. Indeed, many genes required for brain wiring do not encode the guidance receptors themselves, but rather encode parts of the cell biological machinery that governs their spatiotemporal signaling dynamics. For example, the vesicular ATPase is an intracellular sorting and acidification complex involved in regulating guidance receptor turnover and signaling. The protein V100 is a member of this v-ATPase complex, and in this study we show that mutations in the *v100* gene cause brain wiring defects specifically in the adult brain. We further describe a V100-dependent intracellular “sort-and-degrade” mechanism that is required in neurons, and find that when this mechanism is perturbed, it leads to progressive build-up of and aberrant signaling by guidance receptors. These data suggest that a *v100*-dependent neuronal degradation mechanism provides a cell biological basis for guidance receptor turnover and spatiotemporally controlled dynamics during neural circuit formation.

## Introduction

Axon guidance, target selection, and synapse formation determine the neuronal connectivity of the brain and rely on the spatially and temporally controlled localization of guidance receptors [1],[2]. The dynamic localization of these receptors is at least partly regulated at the level of vesicular membrane trafficking through the secretory pathway, endosomal recycling, and endolysosomal degradation [3],[4]. Endosomal routing is also a means of receptor activation and inactivation: receptors may signal from the plasma membrane or endosomal compartments, and receptor signaling can be turned off by endolysosomal degradation [4],[5]. A growth cone may reuse a number of guidance receptors through cycles of endo- and exocytosis. Alternatively, constitutive synthesis and degradation may provide a constant stream of receptors that can be sorted to exert spatiotemporally defined roles. However, for most cell types it is unknown which mode of receptor trafficking prevails to regulate receptor (de)activation during development and function. Similarly, surprisingly little is known about the neuron-specific molecular mechanisms that underlie guidance receptor trafficking for either strategy during brain wiring.

The *Drosophila* nervous system has proven to be a powerful system for the characterization of the molecules that guide axons along their pathways and enable correct target selection [6]–[9]. The visual system has been particularly useful, because both photoreceptors and visual interneurons are dispensable for viability and are easily genetically manipulated in otherwise wild-type flies. Genetic screens based on methods that generate mutant visual neurons in heterozygous flies led to the discovery of numerous important secreted and membrane-associated guidance molecules and receptors, their regulators, and signal-transducing proteins [10]–[12]. Amongst the many known guidance molecules and receptors implicated in visual system development are the cadherins N-Cadherin (N-Cad) and Flamingo (Fmi) [10],[13],[14], the tyrosine phosphatases DPTP69D and Dlar [15]–[17], and the immunoglobulin superfamily cell adhesion molecules Fasciclin 2 (Fas2) and Roughest (Rst) [18]–[20]. Although spatiotemporally dynamic expression has been shown for most of these receptors, almost nothing is known about their intracellular trafficking, activation, turnover, and degradation.

Genetic mosaic screens in the *Drosophila* visual system have also led to the discovery of numerous mutants with membrane and organelle trafficking defects [21]–[23]. The *Drosophila* gene *v100* was originally identified in a screen for mutants that affect synapse formation, specification, or function [23],[24]. *v100* encodes subunit a1 of the V0 complex, the membrane-bound sector of the two-sector vesicular (v-)ATPase [25],[26]. V100 is a neuron-specific subunit of the v-ATPase that is required for neurotransmitter release [24] and provides a neuronal degradation mechanism in photoreceptors. This degradation mechanism is created by a dual function: V100 sorts vesicles into endosomal compartments and subsequently acidifies degradative compartments as part of the v-ATPase holoenzyme. Loss of *v100*-dependent degradation leads to adult-onset degeneration, but no developmental or synaptic specification defects in photoreceptors [27]. Similarly, *v100* mutant embryos exhibit normal nervous system morphology [24].

In this study, we report that a neuron-specific, *v100-*dependent membrane sorting and degradation mechanism is required for brain wiring in *Drosophila*. Loss of *v100* results in missorting and intracellular accumulation of guidance receptors at the time and place where they are subject to active turnover. These accumulations precede axon mistargeting. We further show that guidance receptors aggregate on endolysosomal compartments and cause exacerbated gain-of-function phenotypes in *v100* mutant photoreceptors as well as in the embryonic nervous system. Our findings suggest that continuous receptor turnover and degradation by a neuron-specific mechanism is a general mode of guidance receptor trafficking. Our data further suggest that a *v100*-dependent neuronal degradation mechanism underlies a regulatory strategy that depends on a constant turnover of receptors that can be sorted to exert spatiotemporally defined roles.

## Results

### V100 Is Required for Neuronal Connectivity in the Developing Adult Central Nervous System, but Not in Photoreceptors or Any Embryonic or Larval Neurons

**Figure 1:**
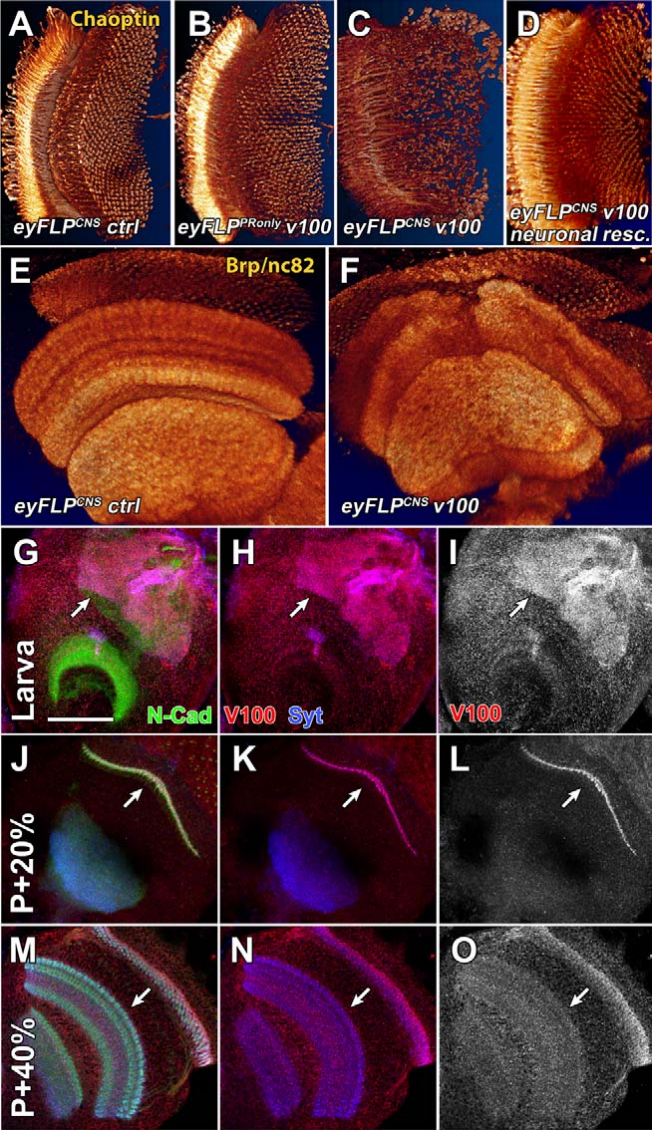
V100 is required for neuronal connectivity in the adult brain. (A–D) 3-D visualizations of photoreceptor projections in the adult brain immunolabeled for Chaoptin (distal to the left). (A and B) Both control and *v100* mutant photoreceptors (*eyFLP^PRonly^ v100*) exhibit wild-type axon targeting patterns. (C and D) In contrast, photoreceptor targeting is disrupted in optic lobes with mutant CNS neurons (*eyFLP^CNS^ v100*) and rescued with pan-neuronal expression of wild-type *v100* cDNA using *elav*-Gal4. (E and F) 3-D visualizations of optic lobe neuropils immunolabeled with Brp/nc82 in control (E) and *eyFLP^CNS^ v100* (F). See also the schematic of optic lobe structure in Figure S1C. (G–O) Characterization of wild-type V100 expression dynamics in the developing optic lobe. (G–I) Wild-type L3 larval brain hemisphere. Developing synapses are immunolabeled with anti-N-Cad (green); active synapses are labeled with anti-Syt (blue); anti-V100 is in red. Note that V100 mostly localizes to active neuropil regions in the larval brain (arrows). (J–L) At P+20%, V100 is strongly enriched in the lamina plexus, i.e., the developing first optic neuropil. (M–O) At P+40%, V100 is enriched in all synaptic neuropils of the developing optic lobe (arrows), where it remains throughout adulthood. Scale bar in (G) for (G–O): 50 µm.

Several genetic mosaic methods have been developed that render visual system neurons homozygous mutant in heterozygous flies [11],[12],[28]. In our previous studies of *v100* function in photoreceptors, we used the “*ey3.5Flp*” system developed by Salecker and colleagues, which renders only photoreceptors mutant [28],[29]. Our studies on *v100* in photoreceptors uncovered defects in neurotransmission [24] and neurodegeneration [27], but no developmental defects. In contrast to this photoreceptor-specific method, the original *eyFLP* system [11] generates thousands of homozygous mutant neurons in the central nervous system (CNS) in addition to photoreceptors. Importantly, *eyFLP* affects only CNS neurons of the visual and olfactory systems that are not required for viability of the organism under laboratory conditions and thereby allows the investigation of *v100* mutant central brain neurons in a living fly (Figure S1) [29]. Surprisingly, we found severe axon pathfinding and targeting defects in these *eyFLP v100* brains that were not present in our previous experiments when only photoreceptors were mutant (Figure 1A–1D). Further analysis of the *eyFLP v100* brain with the active zone marker Brp (nc82) revealed severe structural defects in the arrangement of synaptic neuropils resulting from defective axon pathfinding during pupal development prior to synaptogenesis (Figure 1E and 1F). For clarity we will hereafter refer to the photoreceptor-specific system as *eyFLP^PRonly^* and the original *eyFLP* system that additionally renders CNS neurons mutant as *eyFLP^CNS^*.

Neuron-specific expression of *v100* with the *elav^c155^*-Gal4 driver is sufficient to rescue viability in *Drosophila* [24]. However, Peri and Nusslein-Volhard recently reported a function for the zebrafish ortholog of *v100* in phagosomal/lysosomal fusion in microglial cells [30]. The zebrafish *v100* (atp6v0a1) is a true ortholog because the protein is more closely related to *Drosophila* V100 (61% identical) and the human subunit a1 (82% identical) than it is to the closest paralog in zebrafish (V0 subunit a2, 54% identical). We therefore wondered whether the developmental CNS defects described here could be attributed to a non-neuronal cell type. We analyzed *v100* mutant brains rescued with only neuronal *v100* expression. As shown in Figure 1D, neuronal expression of *v100* rescues the wiring defect of an *eyFLP^CNS^* brain. Hence, *v100* is required in CNS neurons for brain wiring in *Drosophila*.

We have recently shown that V100 is expressed in the pupal and adult visual system [27]. To determine the onset of V100 expression in the developing CNS, we performed co-labeling experiments with the developing synapse marker N-Cad and the active synapse marker Synaptotagmin (Syt). As shown in Figure 1G–1I, anti-V100 labeling of a larval brain hemisphere reveals strong enrichment in the synaptic neuropils of the functional larval brain (arrows). In contrast, regions of neuronal and glial differentiation are labeled at only background levels, suggesting no prominent role during early brain development. However, at the time of axon targeting at 20% pupal development (P+20%), V100 is strongly enriched in the developing first synaptic neuropil in the optic lobe, the lamina plexus, where axon terminals are actively sorting to generate a precise visual map (arrows in Figure 1J–1L). V100 labeling at this time is most prominent in the lamina plexus, but increases in all neuropils throughout development (Figure 1M–1O). Note that V100 labeling, although enriched in the synaptic neuropils, appears distinctly different from that of the synaptic vesicle marker Syt (Figure 1M–1O). These data show that V100 is enriched in specific synaptic regions of the visual system prior to synaptogenesis. Taken together our data indicate that *v100* plays a hitherto unrecognized developmental role in CNS neurons of the adult brain.

Next, we asked whether the observed brain wiring defects are caused by early cell death. Immunolabeling of activated Caspase-3 [31] in *eyFLP^CNS^* brains reveals no significant difference in the number of cells undergoing programmed cell death between mutant and wild type during development (Figure S2A and S2B; *eyFLP^CNS^ v100*: 34±10 apoptotic cells per confocal optic lobe section; control: 31±9) or in 10-d-old optic lobes (mutant neurons marked in green, control unmarked; Figure S2C). This is consistent with the previously documented finding of slow adult-onset degeneration in photoreceptors, which causes cells to become unhealthy long after development is complete [27]. These data indicate that the brain wiring defects are not the result of premature cell death.

### Loss of *v100* Causes Guidance Receptor Accumulations in CNS Neurons in the Optic Lobe

Our previous characterization of V100 function revealed roles in synaptic vesicle exocytosis [24] and endolysosomal degradation in neurons [27]. The brain wiring defects described in this study are unlikely to be caused by defects in neurotransmitter release, since we and others have previously shown that neuronal activity, including synaptic vesicle release, is not required for photoreceptor or optic lobe development [23],[32]. In contrast, *v100*'s role in neuronal endolysosomal degradation could potentially be required for development since many signaling molecules are regulated through the endolysosomal pathway. This idea raises the question how a defect in endolysosomal trafficking could lead specifically to neuronal connectivity defects in the brain without affecting earlier stages of neuronal development.

**Figure 2:**
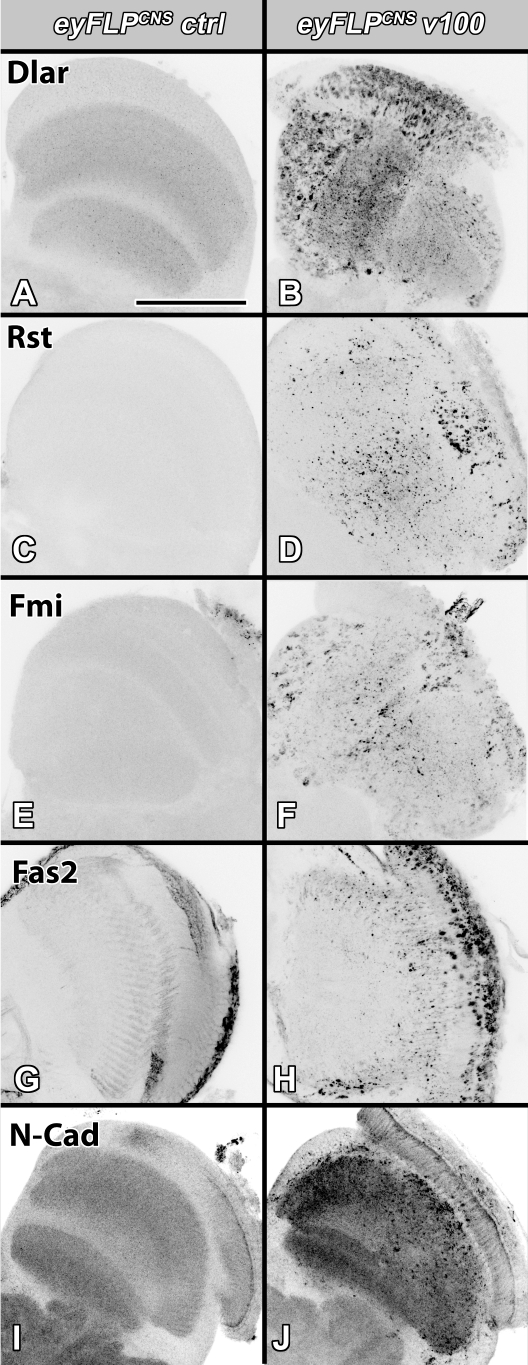
Loss of *v100* causes guidance receptor accumulations in CNS neurons in the optic lobe. Confocal images of 1-d-old *Drosophila* optic lobe sections labeled with antibodies against the guidance receptors Dlar (A and B), Rst (C and D), Fmi (E and F), Fas2 (G and H), and N-Cad (I and J). All five guidance receptors accumulate in cell bodies and at synapses of *eyFLP^CNS^ v100* optic lobes, while accumulations are absent in control brains. Scale bar in (A) for (A–J): 50 µm.

Cell adhesion molecules that function as guidance receptors are key proteins directing axon pathfinding and targeting. To investigate a possible link between *v100* and the observed brain wiring defects, we analyzed several guidance receptors known to play roles during optic lobe development and visual map formation in the *Drosophila* brain. First, we investigated the localization patterns of the five guidance receptors Dlar, N-Cad, Fmi, Fas2, and Rst [10],[13],[17],[33],[34] in 1-d-old control and *eyFLP^CNS^* brains. All five guidance receptors exhibit a similar phenotype of aberrant accumulations in synaptic neuropils and cell bodies of the *eyFLP^CNS^* optic lobe (Figure 2). This phenotype is most pronounced for Dlar and N-Cad, whose wild-type expression patterns in the optic lobe are restricted to synaptic neuropils (Figure 2A, 2B, 2I, and 2J) [13],[17]. While these findings show that guidance receptor localization is indeed affected in *v100* mutant neurons, they also indicate that the underlying intracellular trafficking defect is not specific to a particular guidance receptor. Hence, our results suggest that the developmental defects are a cumulative effect of the mislocalization of many receptors.

### Guidance Receptors Accumulate on Endosomal Compartments in *v100* CNS Neurons

**Figure 3:**
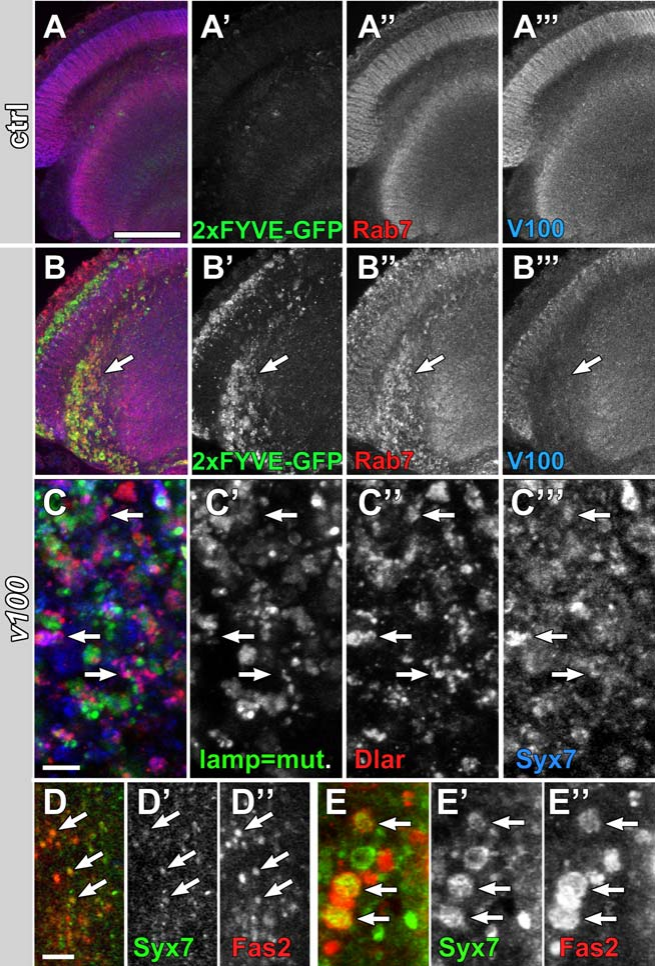
Loss of *v100* in CNS neurons causes endosomal guidance receptor accumulations. (A and B) Confocal sections of adult *eyFLP^CNS^* control (A) and *v100* (B) optic lobes with 50% of all *eyFLP^CNS^* cells labeled with 2xFYVE-GFP. Expression of 2xFYVE-GFP results in a weak 2xFYVE-GFP signal in control cells (A) but strong accumulations in *v100* mutant CNS neurons (B). Similarly, immunolableling of the late endosomal marker Rab7 (red) reveals increased accumulations in *v100* mutant CNS neurons. 2xFYVE-GFP only shown in (A' and B'), Rab7 only in (A″ and B″), V100 only in (A″' and B″'). (C) High-resolution confocal section of adult *eyFLP^CNS^ v100* optic lobe with 50% of all *eyFLP^CNS^* cells labeled with lamp-GFP. Similar to 2xFYVE-GFP and Rab7, the lysosomal marker lamp-GFP accumulates. However, Dlar accumulations colocalize more with the early endosomal marker Syx7 (arrows, blue) than with Lamp-GFP. Lamp-GFP only shown in (C'), Dlar only in (C″), Syx7 only in (C″'). (D and E) High-resolution confocal sections of *eyFLP^CNS^ v100* mutant CNS neuron cell bodies in the medulla cortex (D) and photoreceptor synapses in the lamina (E). Syx7 only shown in (D' and E'), Fas2 only in (D″ and E″). Scale bar in (A) for (A and B): 50 µm; scale bar in (C): 5 µm; scale bar in (D) for (D and E): 5 µm.

*v100* mutant photoreceptors exhibit a slow accumulation of endolysosomal compartments [27]. Intracellular accumulation of guidance receptors might cause developmental defects in CNS neurons by at least two mechanisms. First, receptors might fail to be transported to the plasma membrane, leading to loss-of-function phenotypes. Second, receptors might fail to endocytose or accumulate on signaling-active endosomal compartments, leading to gain-of-function phenotypes. In contrast to such loss- or gain-of-function effects, accumulation of receptors in signaling-inactive lysosomal compartments should not lead to any receptor-specific defects. We therefore analyzed guidance receptor localization on intracellular compartments using an *eyFLP^CNS^*-based approach where only mutant cells are fluorescently marked (mosaic analysis with a repressible cell marker [MARCM]; [35]). First, we confirmed that *v100* mutant CNS neurons have the same endolysosomal accumulations previously described for photoreceptors [27]. As shown in Figure 3A and 3B, both the early endosomal marker 2xFYVE-GFP and the late endosomal marker Rab7 accumulate in *v100* mutant neurons. 2xFYVE-GFP is a cytosolic probe that predominantly marks early endosomal compartments by associating with PI(3)P-rich membranes [36]. Note that in this experiment only 50% of CNS neurons are mutant and these cells are marked with 2xFYVE-GFP expression. As shown in Figure 3A, 2xFYVE-GFP exhibits only low levels of labeling in wild-type clones. In contrast, *v100* mutant CNS neurons exhibit substantial accumulations (arrows in Figure 3B). Rab7-positive compartments exhibit similar accumulations. However, PI(3)P-rich endosomal accumulations are even more apparent than Rab7 accumulations (compare green and red labeling in Figure 3B). These results are consistent with our previous characterization of endolysosomal accumulations in photoreceptors and indicate that *v100* mutant CNS neurons exhibit the same endolysosomal trafficking problem. As in photoreceptors, early endosomal markers are upregulated strongest [27].

Next, we analyzed subcellular guidance receptor localization. As shown in high-resolution confocal images in Figure 3C, the receptor Dlar accumulates in highly heterogeneous compartments in CNS neuronal cell bodies. In this experiment, we marked the mutant cells with the lysosomal marker lamp-GFP, a transmembrane protein that traffics through the endolysosomal pathway and is quickly degraded in wild type [37]. Co-labeling with the early endosomal marker Syx7 reveals that 68.7% of all Dlar accumulations are Syx7-positive (arrows), but only 29.4% are lamp-GFP-positive. We observed similar results for all guidance receptors (Figure 3D and 3E and data not shown). Amongst these receptors, Fas2 exhibited the strongest colocalization with the early endosomal marker Syx7 both in cell bodies of CNS neurons in the medulla cortex (arrows in Figure 3D) and at photoreceptor synapses in the lamina (arrows in Figure 3E). Interestingly, our high-resolution analyses of subcellular localization revealed a pattern of increased guidance receptor accumulations on the outside of large (up to 5 µm) Syx7-positive compartments, as shown in Figure 3E for Fas2. We made similar observations for guidance receptor accumulations using two additional genetic manipulations, namely, increased sorting into endosomal compartments and receptor overexpression in *v100* mutant neurons, as described below. In summary, our findings indicate that guidance receptors accumulate after endocytosis in the compartments most prominently labeled by early endosomal markers.

### Differential Onset of Guidance Receptor Accumulations in CNS Neurons versus Photoreceptors Correlates with the Occurrence of Developmental Defects

**Figure 4:**
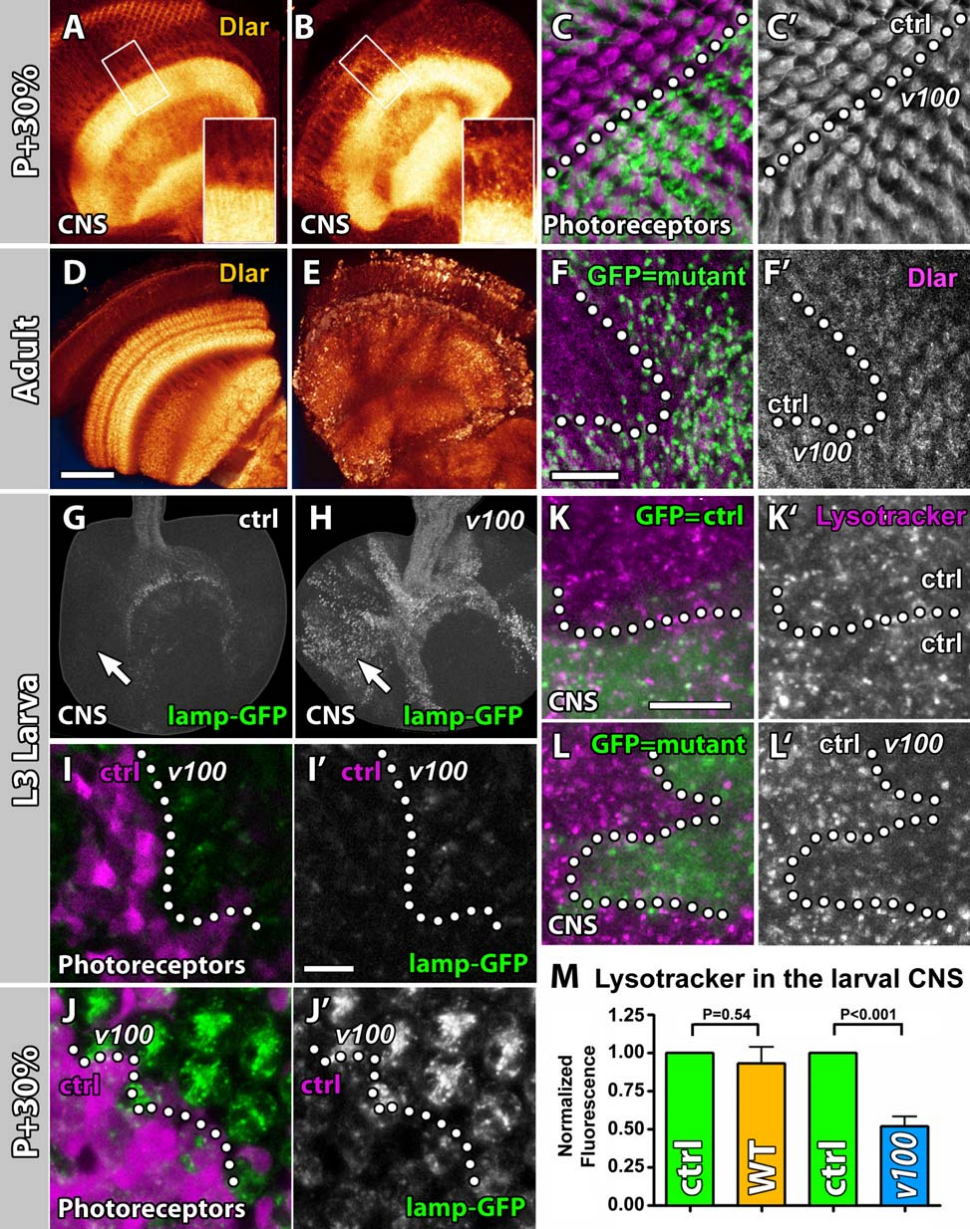
Differential onset of guidance receptor accumulations in CNS neurons versus photoreceptors correlates with the occurrence of developmental defects. (A and B) Confocal sections of P+30% optic lobes immunolabeled with Dlar. (A) Control. (B) *eyFLP^CNS^ v100*. Insets are magnifications of the boxed regions and reveal Dlar accumulations in the mutant neurons that are absent in control. (C) P+30% photoreceptor terminals in the first optic neuropil (lamina plexus). 50% of photoreceptors are mutant and marked with GFP (*eyFLP^PRonly^* MARCM). Note that Dlar (magenta, single channel in [C']) is indistinguishable in mutant and control terminals. The dotted line indicates clonal boundary. (D and E) Confocal sections of adult optic lobes immunolabeled with Dlar. (D) Control. (E) *eyFLP^CNS^ v100*. (F) *eyFLP^PRonly^* MARCM as in (C) reveals mildly increased Dlar levels in mutant terminals (single channel in [F']). The dotted line indicates clonal boundary. (G and H) L3 larval hemispheres of *eyFLP^CNS^* control MARCM (G) and *eyFLP^CNS^ v100* MARCM (H). Only cells rendered homozygous for a wild-type chromosome (G) or the *v100* mutant chromosome (H) express lamp-GFP. Note the strong accumulation of lamp-GFP in mutant CNS neurons (arrows). (I) Clone of *v100* mutant cells in the larval eye disc (wild-type cells marked with RFP [magenta]). Single lamp-GFP channel in (I') and clonal boundary marked with a dotted line. Note that the mutant cells in the eye disc show only very mild lamp-GFP accumulations. (J) Same as in (I) at P+30%. lamp-GFP accumulations are now prominent. (K and L) Live Lysotracker labeling of CNS neurons in *eyFLP^CNS^* MARCM optic lobes. Cells homozygous for a wild-type chromosome in (K) and mutant for *v100* in (L) are marked with GFP. Dotted lines mark clonal boundaries. Note the reduction of Lysotracker signal in the mutant larval CNS neurons. (M) Quantification of Lysotracker measurements. Error bars are standard error of the mean; *n* = 6. Scale bar in (D) for (A, B, D, E, G, and H): 50 µm; scale bar in (F) for (C and F): 10 µm; scale bar in (I') for (I and J): 10 µm; scale bar in (K) for (K and L): 10 µm.

Why do *eyFLP^PRonly^* adult photoreceptors lack a developmental defect? Photoreceptors conclude axon pathfinding less than 48 h after differentiation, while many adult CNS neurons adopt the neural fate many days before brain connectivity is established [38]. *v100* mutant neurons exhibit a progressive increase of intracellular accumulations because of lack of degradation [27]. We reasoned that in CNS neurons, disruptive intracellular accumulations might occur sufficiently early during neuronal development to cause developmental defects. In contrast, in photoreceptors such defects might occur only after the critical developmental time periods of axon pathfinding and target recognition. To compare the time course of intracellular trafficking defects in *eyFLP^PRonly^* and *eyFLP^CNS^*, we investigated guidance receptor localization in developing and adult brains. As shown in Figure 4A and 4B, optic lobes of *eyFLP^CNS^ v100* brains at P+30% exhibit Dlar accumulations that are absent in *eyFLP^CNS^* control brains. To identify even small changes of Dlar levels in photoreceptors, we analyzed mutant and neighboring control terminals in MARCM clones. As shown in Figure 4C, mutant photoreceptors exhibit Dlar levels indistinguishable from control. In 1-d-old adult *eyFLP^CNS^* optic lobes, Dlar accumulations are further increased (Figure 4D and 4E), while mutant photoreceptor terminals are just beginning to show receptor accumulations (Figure 4F). We observed similar temporal dynamics for the other guidance receptors, although with varying onset, localization, and severity of accumulations, as discussed in the next section. Our data show that guidance receptor accumulations occur in both CNS neurons and photoreceptors. However, the photoreceptor defects are delayed and seem to occur sufficiently late to allow normal development. These observations are consistent with the idea that cell-specific axonal targeting defects depend on the dynamics of a progressive degradation and intracellular accumulation defect.

Next, we tested whether the differential onset of Dlar accumulations in CNS neurons versus photoreceptors reflects a general degradation problem of transmembrane proteins that traffic through the endolysosomal system. We analyzed the time course of lamp-GFP accumulations in developing optic lobe neurons (*eyFLP^CNS^*). In late third instar larvae, lamp-GFP exhibits a prominent degradation and accumulation phenotype in the *v100* mutant CNS (Figure 4G and 4H), while mutant photoreceptors show almost no lamp-GFP accumulations at this stage (Figure 4I). However, accumulations do become apparent in photoreceptors at P+30%, i.e., even before Dlar accumulations become discernible (Figure 4J; compare to Figure 4C). In summary, accumulations of lamp-GFP, like accumulations of Dlar, reveal a progressive intracellular degradation defect that occurs earlier in mutant CNS neurons than in photoreceptors.

We have previously shown that *v100* mutant photoreceptors have an acidification defect as evidenced by Lysotracker labeling experiments [27]. Lysotracker is a membrane-permeable dye that accumulates in highly acidified compartments in cells, i.e., lysosomes, late endosomes, and autophagosomes. Larval eye discs show no difference in Lysotracker uptake in mutant versus control cells, while pupal eye discs show a 50% reduction in Lysotracker signal in mutant cells [27]. To characterize the onset of acidification defects in optic lobe CNS neurons, we generated GFP-labeled *v100* mutant clones as before (*eyFLP^CNS^* MARCM). In control experiments, we used the same approach, except both the marked and unmarked cells were wild type. As shown in Figure 4K–4M, we found a significant reduction of Lysotracker signal in mutant CNS neurons of the third instar larva, i.e., at the same time as when photoreceptors are differentiating and do not yet exhibit Lysotracker defects. Furthermore, the strength of the larval CNS defect is reminiscent of photoreceptors at P+40%, i.e., approximately 2 d later. As was the case for Dlar and lamp-GFP accumulations, the observed reduction of Lysotracker-positive compartments in optic lobe CNS neurons is sufficiently early to account for the brain wiring defects. In contrast, a similar reduction of strongly acidified compartments in photoreceptors is observed only after axon pathfinding and visual map formation are concluded.

### Selective Rescue of *v100*-Dependent Sorting into Degradation-Incompetent Compartments Accelerates Developmental Defects

Our data show that both the accumulation of membrane proteins and the loss of Lysotracker-positive degradative compartments precede the onset of developmental defects in CNS neurons. In contrast, our results argue that *v100* mutant photoreceptors lack a developmental defect because endolysosomal defects are delayed. To test the causality of this correlation, we designed an experiment to accelerate *v100* endolysosomal trafficking defects and assay the effect on photoreceptor development. We have previously generated a mutant version of *v100* that accelerates and thereby exacerbates null mutant phenotypes by selectively rescuing the sorting of cargo into degradation-incompetent compartments. Selective rescue of the endosomal sorting function but not the acidification function of *v100* is achieved by expressing the mutant *v100^R755A^* in *v100* null mutant neurons. In contrast, *v100^R755A^* expression in wild-type neurons has almost no effect since the wild-type protein is present to acidify degradative compartments [27].

**Figure 5:**
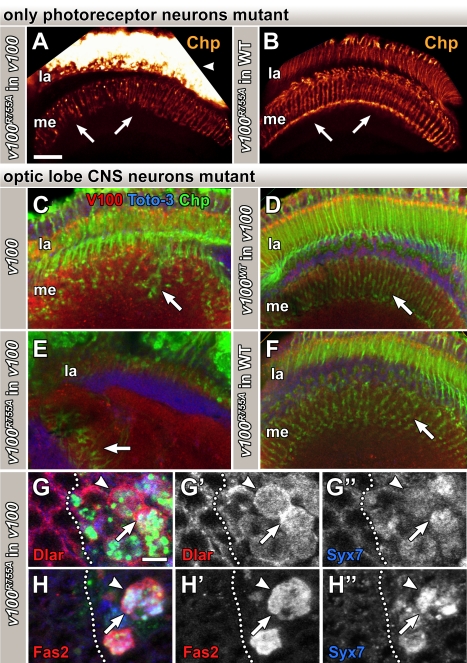
Selective rescue of *v100*-dependent sorting into degradation-incompetent compartments accelerates developmental defects. (A and B) Chaoptin immunolabeling of photoreceptor projections in adult optic lobes. Expression of *v100^R755A^* in *eyFLP^PRonly^ v100* photoreceptors (A) causes targeting defects (arrows) and Chaoptin accumulations (arrowhead) that are absent when *v100^R755A^* is expressed in wild-type (WT) photoreceptors (B). (C–F) Confocal images of *Drosophila* optic lobe sections showing a longitudinal section through the first optic neuropil (lamina) on top and deeper photoreceptor projection below. Green: Chaoptin (photoreceptors); red: V100; blue: Toto-3 (nuclei). Note the targeting defects in *eyFLP^CNS^ v100* in (C), which are dramatically worsened by *v100^R755A^* expression in *v100* mutant neurons (E). In contrast, pan-neuronal expression of *v100* shows full rescue (D), and *v100^R755A^* expression in wild-type CNS neurons causes little or no defect (F). Arrows indicate R7 photoreceptor terminal projections. (G and H) High-resolution confocal sections of 1-d-old adult mosaic eye with wild-type cells to the left and *v100^R755A^* expression in *v100* mutant cells to the right (using MARCM). Shown are the boxed regions of the sections shown at lower resolution in Figure S3A and S3C. Note that the guidance receptors Dlar and Fas2 accumulate both on Syx7-positive compartments (arrows) as well as on the outer membrane (arrowheads). The dotted lines indicate clonal boundaries. Dlar only in (G'), Fas2 only in (H'), Syx7 only in (G″ and H″). Scale bar in (A) for (A–F): 20 µm; scale bar in (G) for (G and H): 5 µm. la, lamina; me, medulla.

As shown in Figure 5A and 5B, *v100^R755A^* expression in *v100* mutant photoreceptors (*eyFLP^PRonly^*) leads to axon targeting defects (arrows in Figure 5A) that are completely absent when *v100^R755A^* is expressed in wild-type neurons, consistent with our previous report that *v100^R755A^* does not act as a dominant-negative [27]. Similarly, these developmental defects are not observed in *v100* null mutant photoreceptors or in mutant photoreceptors that are rescued with wild-type *v100* (Figure 1B and 1D). In addition, large amounts of the photoreceptor-specific transmembrane protein Chaoptin accumulate when *v100^R755A^* is expressed in mutant neurons (arrowhead in Figure 5A). These data suggest that *v100^R755A^* expression in mutant neurons accelerates intracellular accumulations and causes developmental defects in photoreceptors. Next, we assessed the effect of *v100^R755A^* expression on the wiring defect in *v100* mutant optic lobe CNS neurons (Figure 5E). Strikingly, *v100^R755A^* causes a dramatically worse wiring defect than the null mutant and effects a total loss of recognizable neuropil structure (Figure 5C and 5E). Neuronal expression of wild-type *v100* fully rescues this defect (Figure 5D), and no such defect is observed when *v100^R755A^* is expressed in wild-type neurons (Figure 5F).

Where do guidance receptors accumulate in neurons with developmental defects caused by *v100^R755A^*-accelerated sorting? Figure 5G and 5H shows cross-sections through photoreceptor cell bodies of 1-d-old eyes in which the cells on the right side of the clonal boundaries are *v100* mutant and express *v100^R755A^*, while the neighboring clones on the left side are wild type. In this experiment the mutant cells are marked with synapto-pHluorin (green, MARCM), which accumulates in endosomal compartments [27]. As shown for Dlar and Fas2 in Figure 5G and 5H, guidance receptors exhibit strong accumulations in mutant photoreceptor cell bodies containing synapto-pHluorin aggregates. Interestingly, a substantial amount of both Dlar and Fas2 encircles Syx7 labeling and is found on the plasma membrane of the dramatically enlarged cell bodies (arrow heads in Figure 5G and 5H) as well as on Syx7-positive compartments (arrows). Very similar cell body membrane accumulations are observed for the other guidance receptors (data for Dlar, Rst, and Fas2 in Figure S3. These observations suggest an endocytic defect of membrane receptors. While it is at this point unclear whether these endocytic defects are primary or secondary to an accelerated clog-up or recycling problem in the endocytic pathway, these observations clearly show that guidance receptors do not accumulate only in signaling-incompetent lysosomal compartments. In summary, our data indicate that accelerated endolysosomal sorting into degradation-incompetent compartments causes guidance receptor accumulations on plasma and/or endosomal membranes and accelerates the onset and severity of developmental defects in both photoreceptors and CNS neurons.

### Sorting into Degradation-Incompetent Compartments Reveals Different Guidance Receptor Turnover Rates

**Figure 6:**
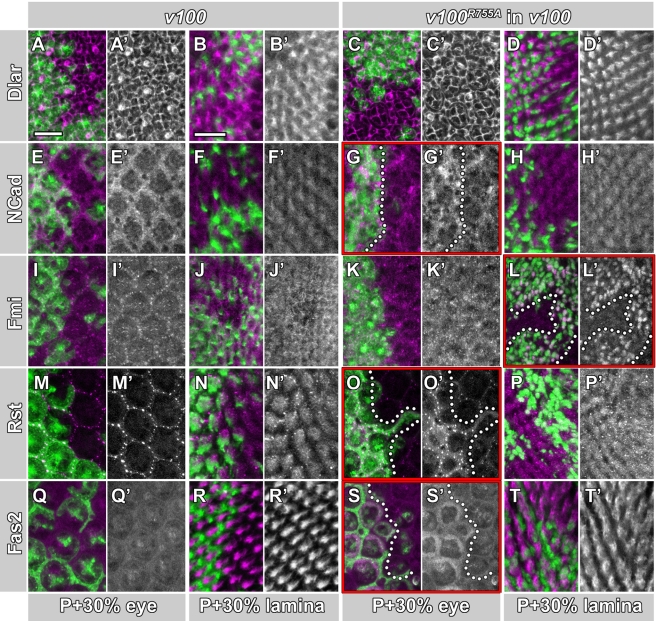
Sorting into degradation-incompetent compartments reveals guidance-receptor-specific turnover rates. Confocal images of developing eye discs and laminae at P+30% using *eyFLP^PRonly^* MARCM. GFP-marked cells in the four left columns are mutant for *v100*. GFP-labeled cells in the four columns on the right express *v100^R755A^* in a *v100* mutant background. Unlabeled cells are wild-type control in all panels. (A–D) Immunolabeling of Dlar (magenta). Neither loss of *v100* (A and B) nor accelerated sorting in degradation-incompetent compartments (C and D) causes obvious Dlar accumulations at P+30% either in the eye (A and C) or at photoreceptor terminals (B and D). Single channels of Dlar immunolabeling shown in (A'–D'). (E–H) Same as (A–D), except immunolabeling of N-Cad (magenta). Note that accelerated sorting into degradation-incompetent compartments with *v100^R755A^* leads to mild N-Cad accumulations in the developing photoreceptor cell bodies (G). (I–L) Same as (A–D), except immunolabeling of Fmi (magenta). Note the strong accumulation of Fmi selectively at photoreceptor terminals expressing *v100^R755A^* in mutant photoreceptors (L). (M–P) Same as (A–D), except immunolabeling of Rst (magenta). Note the strong accumulation of Rst selectively in the cell bodies of the developing eye (O). (Q–T) Same as (A–D), except immunolabeling of Fas2 (magenta). Note the strong accumulation of Fas2 selectively in the cell bodies expressing *v100^R755A^* in (S). Quantification for experiments marked with red boxes: (G) N-Cad shows a 1.24-fold increase (±0.04) in mutant cells compared to control, (L) Fmi shows a 1.47-fold increase (±0.25), (O) Rst shows a 2.18-fold increase (±0.50), and (S) Fas2 shows a 1.81-fold increase (±0.11). Scale bar in (A) for all eye sections: 10 µm; scale bar in (B) for all lamina sections: 10 µm.

Our observations suggest that accelerated sorting by *v100^R755A^* expression in *v100* mutant neurons during brain wiring accelerates the accumulation of guidance receptors. To test this idea we analyzed Dlar, N-Cad, Fmi, Fas2, and Rst in *v100* mutant photoreceptors (*eyFLP^PRonly^*) with or without *v100^R755A^* expression at P+30%. As shown in Figure 6, none of the guidance receptors exhibit obvious receptor accumulations either in the developing eye or at photoreceptor synapses at this developmental time point in the *v100* null mutant (also compare Figure 4C). Very mild increases are only just discernible for Rst in the developing eye and for Fmi at synapses (arrows in Figure 6J' and 6M'). In comparison, accelerated sorting into degradation-incompetent compartments (*v100^R755A^* in *v100*) leads to increased accumulations with highly variable severity and in different parts of the neuron for these five receptors at P+30%. As shown in Figure 6O, Rst accumulations are strongly increased in the eye, while N-Cad and Fas2 exhibit comparably mild increases (Figure 6G and 6S) and Dlar and Fmi are apparently unaffected in cell bodies in the eye (Figure 6C and 6K). In contrast, at photoreceptor synapses in the same brains, Fmi is strongly increased (Figure 6L), whereas Rst, Dlar, N-Cad, and Fas2 show mild or no increased accumulations (Figure 6D, 6H, 6P, and 6T). Since all five guidance receptors analyzed here as well as other transmembrane proteins including lamp-GFP and CD8-GFP accumulate in *v100* mutant neurons over time (compare Figures 2 and 4), we conclude that only receptors that are in the endolysosmal system at a given time in the cell body or at the synapse are subject to *v100^R755A^*-accelerated sorting and *v100*-dependent degradation. This interpretation is consistent with the two strongest effects shown here: Rst plays a key role in membrane sorting during eye development at P+30%, but is not yet strongly expressed at synapses [20],[39], whereas Fmi plays a key role in photoreceptor targeting at P+30% [13]. Co-labeling of the *v100^R755A^*-accelerated accumulations of Rst in the eye and Fmi at synapses with Syx7 reveals many colocalizing accumulations (Figure S4). The colocalization with the early endosomal marker is consistent with the findings for both *v100* mutant photoreceptors and CNS neurons (Figures 3C–3E, 5G, and 5H). In summary, specifically restoring the sorting function of *v100* accelerates the rate of guidance receptor accumulation in developing neurons and reveals the spatiotemporal dynamics of guidance receptor turnover.

### Guidance Receptors Accumulate in Signaling-Competent Compartments in *v100* Mutant Photoreceptors

Our findings in both photoreceptors and CNS neurons indicate that guidance receptors accumulate on membranes where they could potentially exert increased signaling. In particular, the *v100^R755A^*-accelerated sorting leads to accumulations of receptors both on endosomal compartments and on the plasma membrane. These findings are not consistent with the idea of accumulations in signaling-incompetent lysosomal compartments or failed exocytic membrane delivery. Rather, our data strongly suggest defects along the endocytic pathway.

**Figure 7:**
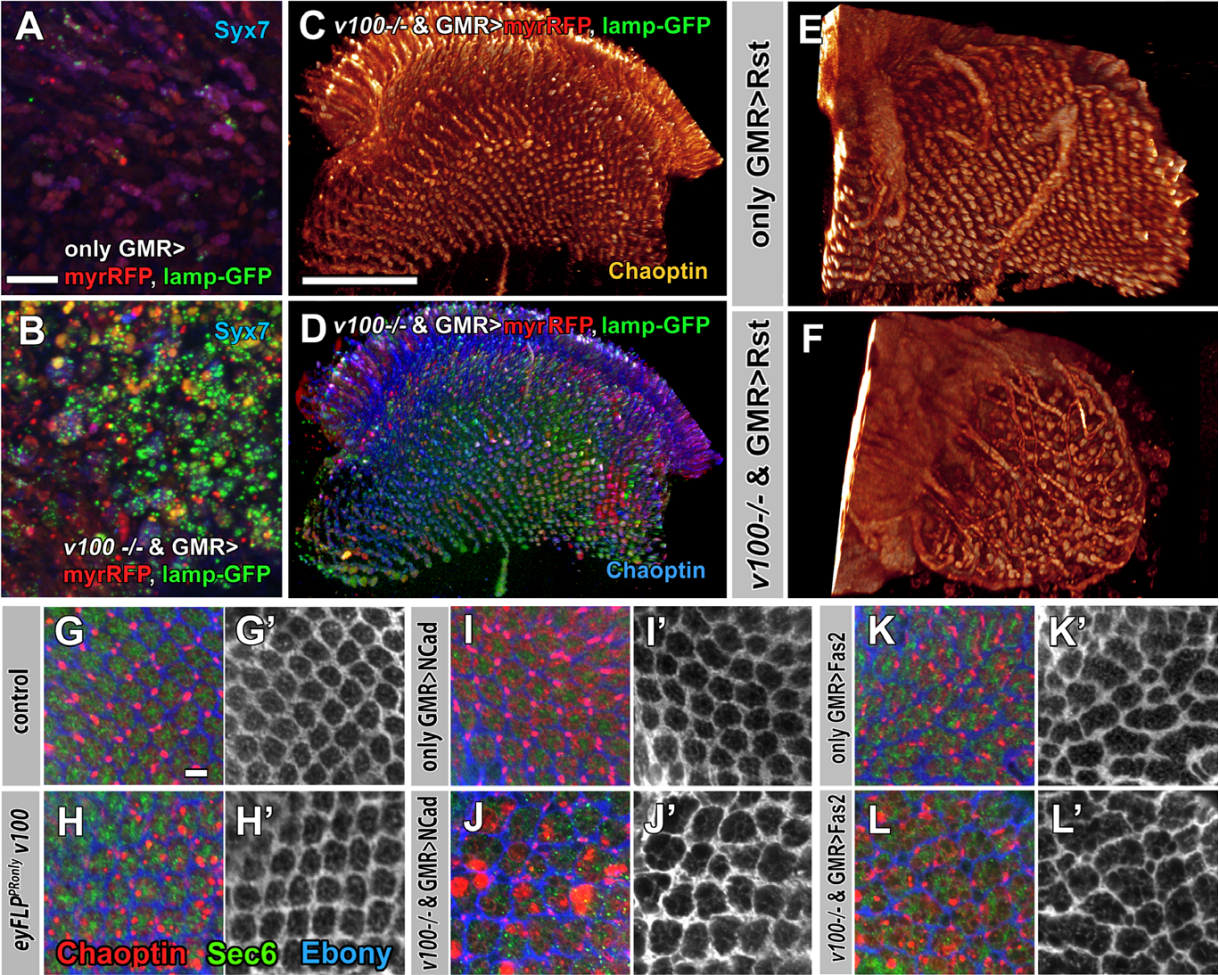
Guidance receptors accumulate in signaling-competent compartments in *v100* mutant photoreceptors. (A and B) Adult lamina cross-sections of control (A) and *eyFLP^PRonly^ v100* photoreceptor terminals (R1–R6). (B) Photoreceptor-specific expression of myristoylated RFP (red) and Lamp-GFP (green); blue: Syx7. (C and D) 3-D visualizations of R7 and R8 axonal projections of Chaoptin-labeled photoreceptors mutant for *v100* and co-overexpressing both myrRFP and Lamp-GFP. Note that both proteins strongly accumulate, but do not cause developmental defects. Single channel in (C). (E and F) 3-D visualizations of photoreceptor projections expressing the guidance receptor Rst in wild-type photoreceptors (E) and *v100* mutant photoreceptors (F). The increase in axon fasciculations and pathfinding defects is a specific exacerbation of the Rst gain-of-function phenotype. (G–L) Confocal images of adult lamina cross-sections labeled with Chaoptin (R1–R6 photoreceptors, green), Sec6 (interneurons, red), and Ebony (glia, blue). Control (G) and *eyFLP^PRonly^ v100* (H) show normal lamina structure. Photoreceptor-specific expression of the guidance receptor N-Cad has no effect in control (I) but disrupts pattern formation in *v100* mutant photoreceptors (J). Fas2 expression causes mild developmental defects in control (K) that worsen in the mutant (L). Ebony only in (G'–L'). Scale bar in (A) for (A and B): 10 µm; scale bar in (C) for (C–F): 50 µm; scale bar in (G) for (G–L): 5 µm.

To directly test the activity of missorted guidance receptors in *v100* mutant neurons, we designed an experiment to challenge *v100* mutant photoreceptors (*eyFLP^PRonly^*) with overexpression of guidance receptors and other transmembrane cargo. We reasoned that increased numbers of guidance receptors should lead to receptor-specific gain-of-function phenotypes that are exacerbated when *v100*-dependent sorting and degradation are removed. In contrast, increased numbers of membrane proteins without signaling function should not cause developmental defects, even though they may still accumulate in the same intracellular compartments. As control transmembrane cargo, we selected lamp-GFP and myristoylated RFP (myrRFP). Overexpression of both lamp-GFP and myrRFP leads to pronounced accumulations in synaptic terminals of *eyFLP^PRonly^ v100* mutants, but not in the synaptic terminals of wild-type photoreceptor neurons (Figure 7A and 7B). However, even co-overexpression of both transmembrane-anchored fluorescent probes in *v100* mutant photoreceptors causes no appreciable developmental defects (Figure 7C and 7D). We conclude that accumulations of membrane proteins without signaling function are not sufficient to cause developmental defects.

In contrast, overexpression of Rst, Fas2, or N-Cad in *v100* mutant photoreceptors causes well-defined, strong axon pathfinding or visual map formation defects. Specifically, overexpression of Rst causes distinct axon fasciculation and pathfinding defects, a phenotype that is dramatically worsened in a *v100* mutant background (Figure 7E and 7F). In contrast, overexpression of N-Cad in wild-type photoreceptors does not cause any appreciable developmental defect, whereas overexpression of N-Cad in *v100* mutant photoreceptors causes distinct defects in visual map formation in the lamina (Figure 7G–7J). This phenotype is very different from the misrouted axon bundles caused by increased Rst function. Whereas the Rst-specific fasciculation and pathfinding defects are best shown in the 3-D visualizations of axon projections in the brain, the visual map formation defect in the lamina is best demonstrated by the lamina cross-sections shown in Figure 7G–7J. Similarly, overexpression of Fas2 in wild-type photoreceptors causes no pathfinding defects, but a highly specific sorting defect of synaptic terminals in the lamina (i.e., a specific visual map formation defect; 11% of synaptic cartridges contain more than eight or less than four terminals, compared to <1% in wild type), and this Fas2-dependent phenotype is substantially worsened in a *v100* mutant background (23% of synaptic cartridges contain less than four or more than eight terminals; Figure 7K and 7L). In contrast, loss of Fas2 in photoreceptors causes no obvious defects in axon targeting or visual map formation (data not shown). These results show that overexpression of guidance receptors, but not membrane-tagged fluorescent probes without signaling function, causes specific developmental defects that strongly suggest exacerbated gain-of-function phenotypes.

Our findings are consistent with the idea that both guidance receptor overexpression and increased receptor sorting into degradation-incompetent compartments lead to developmental defects because of increased guidance receptor activity. Indeed, both genetic manipulations lead to increased colocalization of guidance receptors with the early endosomal Syx7, as shown for Fas2 in Figure S5. Taken together with the finding of early accumulations of guidance receptors on endosomal compartments in *v100* mutant CNS neurons, our findings support the idea that brain wiring defects in the adult CNS result at least partially from increased guidance receptor activity.

### V100-Dependent Guidance Receptor Accumulations Cause Gain-of-Function Defects in the Embryo

**Figure 8:**
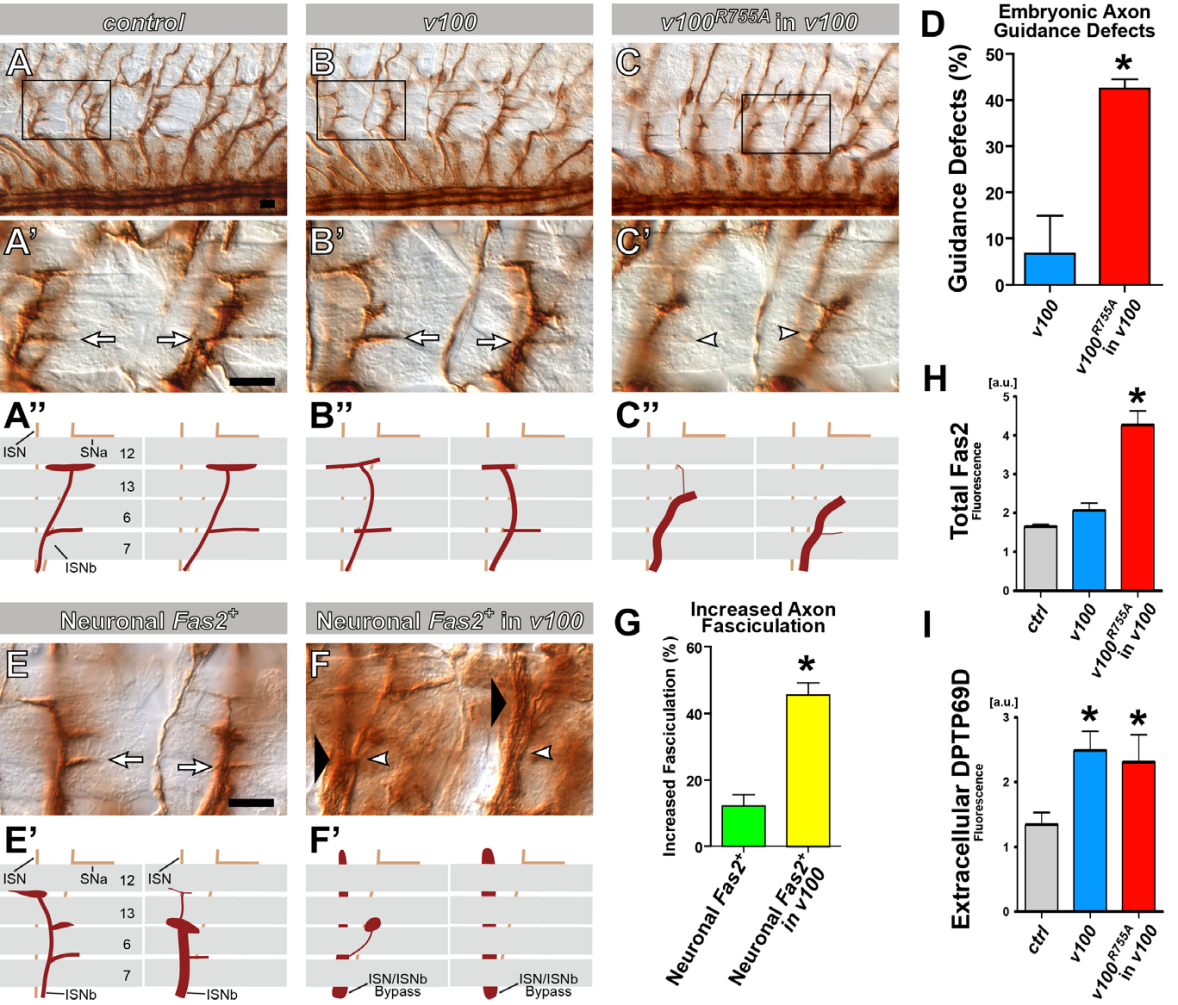
V100-dependent guidance receptor accumulations cause gain-of-function defects in the embryo. (A–C, E and F) Photomicrographs of filleted *Drosophila* embryos stained with the motor axon marker 1D4 (anti-Fas2) where normal innervation (arrows), abnormal innervation (white arrowheads), and increased axon-axon fasciculation “bypass” defects (large black arrowheads) are demarcated. (A–C) Motor axons within the ISNb axon pathway innervate muscles 6 and 7 normally in control (*elav*-Gal4 only) (A) and *v100* (B) embryos but show abnormal guidance and targeting in *elav*> *v100^R775A^*; *v100* (C) embryos. Boxed regions are shown at higher magnification (A'-C') and are also depicted schematically (A″–C″). (D) The percentage (%) of abnormal muscle 6/7 innervation is shown (normalized to control levels; *n*>60 hemisegments/genotype) and reveals a statistically significant increase in guidance defects when there is accelerated sorting into degradation-incompetent compartments (*v100^R775A^* is expressed in the *v100* mutant background). (E–G) ISNb motor axons were examined in embryos expressing one copy of the Fas2 transgene in all neurons in either a wild-type (E) or a *V100* mutant (F) background, and the results reveal that loss of *v100* increases the percentage (%) of abnormal Fas2-mediated ISNb axonal fasciculation events (bypass with the ISN or stall phenotypes; [33]; *n*>140 hemisegments/genotype). (H) Quantification of Fas2 fluorescence in the embryo for the data shown in Figure S6A–S6C. Note that this panel is identical to the one in Figure S6G. a.u., arbitrary units. (I) Quantification of detergent-free immunolabeling of extracellular guidance receptor DPTP69D at the neuromuscular junction. See also images in Figure S7. Asterisks denote statistical significance with *p*<0.01. Scale bar in (E) for(A–C, A'–C', E, and F): 10 µm. Error bars are standard error of the mean; *p*-value by Student's *t* test.

To further test the idea that *v100*-dependent accumulations of guidance receptors lead to increased receptor signaling we turned to the *Drosophila* embryonic nervous system. *Drosophila* embryonic motor axons have long provided a simple in vivo model for characterizing axon guidance molecules [7],[40], since individual axons can be followed to their targets and phenotypes that result from increased signaling can often be differentiated from loss-of-function defects (e.g., [19],[33],[41],[42]). The discovery of the progressive *v100*-dependent neuronal degradation mechanism makes clear predictions for guidance receptor sorting in the embryonic nervous system. Specifically, we propose that 24 h of embryonic development is not sufficient to lead to aberrant receptor function. However, both accelerated sorting into degradation-incompetent compartments (*v100^R755A^* in *v100*) as well as guidance receptor overexpression in *v100* mutant neurons should accelerate the occurrence of receptor-specific phenotypes similar to the effects shown for photoreceptors. To test this hypothesis, we analyzed axon pathfinding and guidance receptor sorting in the embryo. The guidance receptor Fas2 not only plays a critical role in axon pathfinding, but also is one of the most commonly used markers to analyze pathfinding, branching, and fasciculation defects in the embryonic nervous system [40]. Furthermore, the *Drosophila* embryonic nervous system has been used as a model to differentiate the effects of increased versus decreased Fas2 signaling [19],[33],[43]. As shown in Figure 8A and 8B, Fas2-positive ISNb axons reveal no statistically significant guidance defects in null mutant embryos (blue bar in Figure 8D). In contrast, accelerated sorting into degradation-incompetent compartments (*v100^R755A^* in *v100*) leads to statistically significant axon guidance defects (Figure 8C; red bar in Figure 8D). Interestingly, these phenotypes are indicative of increased axon-axon fasciculation, a phenotype that is known to result from increased Fas2 signaling in axons [33]. As shown in Figure 8H, Fas2 immunolabeling is significantly increased in *v100^R755A^*-“rescued” embryos. Furthermore, co-labeling with the early endosomal marker Syx7 reveals increased accumulations of Fas2 in degradation-incompetent compartments (*v100^R755A^* in *v100*) of embryonic neurons (Figure S6A–S6C, S6G, and S6H). Similar to *v100^R755A^* expression in *v100* mutant neurons, overexpression of Fas2 in *v100* mutant neurons leads to significantly enhanced gain-of-function fasciculation defects compared to Fas2 overexpression in *v100* heterozygous or wild-type neurons (Figure 8E–8G). These results reveal that the V100-dependent degradation pathway regulates the levels of Fas2 in neurons in both the *Drosophila* visual and embryonic systems, and strongly argue that these *v100*-dependent accumulations lead to increased Fas2 signaling. Interestingly, certain aspects of embryonic nervous system development and axon pathfinding remain largely unaffected. For example, midline crossing, which is partly regulated by the Slit-Robo system [44], is mostly resistant to *v100^R755A^*-accelerated receptor sorting (with only low-penetrance defects). Similarly, *v100^R755A^*-accelerated receptor sorting does not enhance Sema-1a/PlexA–mediated repulsive signaling at the midline (data not shown; [45]). However, analysis of Robo1 receptor expression reveals mild accumulations in the ventral ganglion that are increased by *v100^R755A^* expression (Figure S6D–S6F, S6I, and S6J). These findings are consistent with our observation that *v100^R755A^* expression in *v100* mutant neurons reveals spatiotemporally specific turnover rates of guidance receptors. A straightforward explanation for the lack of midline crossing defects is that loss of degradation does not lead to aberrant Robo signaling within the time frame of embryonic development.

Finally, the embryonic nervous system allows us to directly test in the *v100* mutant whether guidance receptors are successfully trafficked to the membrane surface through the secretory pathway. We made embryonic filet preparations in which axons are directly accessible to antibody washing solutions in the absence of detergent. Since the Fas2 immunohistochemistry antibody is specific to the intracellular domain, we tested this idea with an antibody against the extracellular domain of the guidance receptor DPTP69D, which functions in ISNb axon pathfinding similarly to Fas2, at the same time and place [46]. As shown in Figures 8I and S7, this receptor exhibits slightly increased levels of expression on the axon membrane surface in *v100* mutant and *v100^R755A^-*“rescued” neurons compared to control. Taken together, our analysis of *v100*-dependent receptor sorting in the embryonic nervous system fully supports our results in photoreceptors and adult brain CNS neurons. Specifically, these results highlight that numerous guidance receptors are subject to the *v100*-dependent “sort-and-degrade” mechanism, that receptor trafficking defects are downstream of receptor secretion in the endosomal pathway, and that increased levels of guidance receptors lead to exacerbated gain-of-function defects.

## Discussion

In this paper, we show that loss of a neuron-specific v-ATPase-dependent degradation mechanism leads to brain wiring defects in *Drosophila*. Neurons mutant for the v-ATPase V0 subunit a1, *v100*, progressively accumulate degradation-incompetent compartments that contain multiple classes of guidance receptors. Both accelerated sorting into degradation-incompetent compartments and overexpression of guidance receptors in *v100* mutant neurons lead to increased receptor accumulations on signaling-competent membranes and accelerate developmental defects in photoreceptors and embryonic motor neurons. However, only accumulations of guidance receptors, but not transmembrane proteins without signaling function, lead to specifically exacerbated gain-of-function defects. Hence, our results indicate that block of *v100*-dependent degradation can lead to the accumulation of guidance receptors in signaling-competent compartments. We conclude that in the *Drosophila* CNS, *v100*-dependent receptor degradation is required during development for the cell to spatiotemporally control guidance receptor signaling, which is in turn necessary for neuronal connectivity in the brain. Our findings suggest that continuous turnover and degradation is a general mode of guidance receptor regulation that sets the stage for other trafficking mechanisms that instructively regulate guidance receptor localization and signaling.

### The Role of *v100*-Dependent Intracellular Trafficking in Neuronal Development

Membrane trafficking underlies the growth and remodeling of axonal and dendritic branches. However, the loss of *v100*-dependent endolysosomal trafficking presented here has no apparent effect on membrane addition and remodeling. Instead, we identified a role for *v100* in intracellular receptor trafficking. Intracellular trafficking and the v-ATPase are known to play critical roles in the dynamic localization and signaling of a plethora of transmembrane receptors [47],[48]. Receptors may signal from the plasma membrane or may be endocytosed to exert a signaling function [5]. A prominent example in neuronal development is the regulation of cellular differentiation by endocytosis of the Notch ligand Delta [49]. However, loss of *v100* causes no early developmental defects, and *v100* is therefore not required for the regulation of receptor-mediated signaling that governs cellular differentiation and early tissue patterning. In contrast, we report that CNS neurons of the developing adult brain exhibit axon pathfinding and synaptic specification defects. Our findings indicate that V100 has a specialized task in neurons and has no function in the essential endolysosomal machinery required for early development. In contrast, the loss of key subunits of the V1 complex of the v-ATPase (which is probably required for all v-ATPase function) cause cell lethality. Specifically, *ey*FLP *vha55* and *ey*FLP *vha68* lead, in stark contrast to *ey*FLP *v100*, to an abolishment of the eye (P. R. H, unpublished data).

The *v100* mutant phenotypes are most similar to those of two other intracellular trafficking mutants that we have described before, *n-syb* mutants and *sec15* mutants. Loss of *n-syb*, the gene that encodes the vesicle SNARE neuronal Synaptobrevin, leads to guidance receptor accumulations and synaptic specificity defects in the *Drosophila* visual system [50]. *sec15* encodes a component of the Exocyst complex required for neuronal targeting or secretion functions other than neurotransmitter release. Similar to loss of *n-syb*, loss of *sec15* leads to mislocalization of guidance receptors and photoreceptor targeting defects [29]. These findings represent mounting evidence for the employment of neuronal intracellular trafficking machinery during brain wiring. However, the neuronal degradation function presented here for *v100* differs from the earlier findings for *n-syb* and *sec15*, in that loss of *v100* does not lead to targeting or “tiling” defects in the photoreceptor terminal field. Curiously, the guidance receptors most prominently affected by loss of either *n-syb* or *sec15* are Fas2 and Dlar, while N-Cad and Fmiare not affected in *sec15* mutant photoreceptors [29]. In contrast, all these guidance receptors are affected by loss of *v100* in CNS neurons. We interpret these differences in the context of differing molecular functions: while loss of *sec15* may lead to targeting defects of a subpopulation of neuronal vesicles required for guidance receptor localization, loss of *v100* disrupts general receptor turnover downstream of the secretory pathway in neurons. This disruption could be partly due to “clog-up” of the endolysosomal pathway or due to defective endosomal recycling.

Our challenge experiments using guidance receptor overexpression in *v100* mutant photoreceptors and embryonic motor neurons are similar to Wingless overexpression experiments in intracellular degradation mutants. Dubois et al. [51] showed that Wingless is targeted to lysosomes and is continuously and specifically degraded posterior to each stripe of Wingless transcription. Disruption of lysosomal degradation leads to Wingless accumulations and, together with Wingless overexpression, ectopic signaling [51]. Similarly, we find that guidance receptors undergo constant turnover (see discussion in the next section) and that their overexpression in *v100* mutant neurons leads to ectopic signaling. For example, N-Cad overexpression in wild-type photoreceptors, analogous to the Wingless experiments, does not cause obvious defects. In contrast, N-Cad overexpression in *v100* mutant photoreceptors causes gain-of-function phenotypes. Similarly, overexpression of low levels (one copy) of Fas2 causes only very mild fasciculation defects in embryonic motor neurons [33]. In contrast, the same level of Fas2 overexpression in *v100* mutant neurons causes a phenotype very similar to high levels of Fas2 overexpression (two copies) [33]. These observations strongly suggest increased gain-of-function phenotypes and are not consistent with loss-of-function phenotypes for these receptors. However, these findings do not exclude the possibility that parts of the compound brain wiring defects in *eyFLP^CNS^ v100* mutants are due to loss of function for other proteins affected by *v100*-dependent sorting.

Importantly, *v100* is a neuron-specific gene, and its loss does not lead to hallmark phenotypes of general lysosomal degradation mutants, including autofluorescent lipofuscin or ceroid accumulations or aberrant multilamellar lysosomal organelles [27],[52],[53]. Hence, V100 provides a neuronal degradation mechanism specifically required after differentiation for late brain development and neuronal maintenance.

### The Role of Receptor Turnover during the Establishment of Synaptic Specificity

How guidance receptors are dynamically localized is unknown for most receptors. Several guidance receptors are known to be regulated by intracellular trafficking. Sema3A-induced endocytosis of Neuropilin-1 has been shown to be required for growth cone collapse during axon guidance [54]. Similarly, internalization of UNC-5A prevents UNC-5A-mediated growth cone collapse in hippocampal axon guidance [55]. One of the best characterized examples of intracellular dynamic sorting is the guidance receptor Robo [44],[56],[57]. During embryonic nervous system development certain axons are prevented from crossing the midline by a repellent guidance cue that binds to the Robo receptor. During a short time window, Robo is removed from the plasma membrane and the axon crosses the midline exactly once. Thereafter, Robo receptors return to the membrane and prevent the axon from crossing again. Remarkably, this dynamic relocalization of the Robo receptor is achieved by diverting a continuous supply of receptors from the endoplasmic reticulum/Golgi temporarily into the endolysosomal pathway for degradation by means of the intracellular sorting receptor Comm. Hence, the dynamic membrane presentation of Robo receptors on the growth cone is not regulated by endo- and exocytosis of a fixed amount of receptors. Instead, the regulation occurs via an intracellular sorting receptor, revealing a strategy that relies on constitutive synthesis and degradation of receptors that can be sorted to exert spatiotemporally defined functions. Notably, the proposed diversion of Robo receptors into degradative compartments is only very short. Indeed, we observe a mild increase of Robo accumulations in embryonic neurons. However, the lack of developmental defects suggests that these accumulations are not sufficient to cause aberrant signaling. We propose that loss of *v100*-dependent degradation leads to only a slow build-up of undegraded receptors, and 24 h of embryo development is not sufficient to lead to neuronal connectivity defects.

The role of *v100* in guidance receptor turnover is most strikingly highlighted by the selective rescue of *v100*-dependent sorting into degradation-incompetent compartments. Rescue of the sorting function, without rescue of acidification-dependent degradation, leads to a dramatically accelerated accumulation of endogenously expressed guidance receptors. Interestingly, these accumulations are increased compared to the *v100* null mutant. Hence, V100 actively promotes vesicle sorting into endosomal compartments destined for degradation. In addition, we observe accumulations of guidance receptors on the plasma membrane. While we cannot exclude a primary defect in endocytosis, a secondary effect due to clog-up of the endolysosomal system or endosomal recycling defects seems more likely. In either case, these observations clearly show that guidance receptors do not exclusively accumulate in signaling-incompetent compartments. In addition, the absence of early developmental defects indicates, and our staining of DPTP69D in the embryo demonstrates, functional guidance receptor exocytosis.

Our findings reveal several key features of *v100*-dependent “sort-and-degrade.” First, in the complete absence of *v100*-dependent sorting and degradation, this turnover is at least partially taken over by a *v100*-independent degradation pathway. This interpretation is consistent with our previous model, in which V100 acts in parallel to an essential endolysosomal pathway that ensures cellular differentiation and viability [27]. Second, the progressive nature of the “sort-and-degrade” mechanism is similar in all different types of neurons analyzed here. We conclude that the occurrence of neuronal connectivity defects is a function of the duration between neuronal differentiation and synaptic specification. Third, these experiments reveal that guidance receptors are subject to a constant turnover. Indeed, combined measurement of guidance receptor accumulation in *v100* mutant neurons and *v100^R755A^*-“rescued” neurons is a tool to assess the turnover rate of different guidance receptors. The idea that there is constant turnover is supported by the observation of different accumulation kinetics for several guidance receptors investigated here. For example, our experiments at P+30% reveal high Rst turnover in the developing eye but not at synapses, high Fmi turnover at synapses but not in the eye, and very little Dlar turnover at this developmental time point. Taken together, our findings suggest that *v100*-dependent “sort-and-degrade” is required for guidance receptor turnover, and its manipulation is a method to assess receptor turnover at different time points.

## Materials and Methods

### *Drosophila* Strains and Conditions of Culture

*y w; P*(*ry*+ = *neo* FRT82B) isogenized flies were used as control animals. *v100* null mutant and overexpression lines have previously been described [24]. Allele *v100^4^* was the mutant allele used in all experiments. All further fly strains are described in detail below. Flies were reared at room temperature, except for pupal staging experiments, where flies were reared at 25°C (P+100% corresponds to 103 h).

### Mosaic Analyses

For photoreceptor-specific mosaics (*eyFLP^PRonly^*) [28],[29] the base genotype is *ey3.5FLP*;;FRT82B,*v100*/FRT82B,cl,w+. For optic lobe CNS neuron clones (*eyFLP^CNS^*) [11] the base genotype is eyFLP;;FRT82B,*v100*/FRT82B,cl,w+. In order to express different reporters in either photoreceptors or all neurons, the following flies were generated. (1) For *eyFLP^PRonly^*: *ey3.5FLP*;GMR-Gal4,(X*); FRT82B,*v100*/FRT82B,cl. (2) For *eyFLP^CNS^*: *eyFLP*,*elav*-Gal4;(X*); FRT82B,*v100*/FRT82B,cl. (X*) stands for one of the following UAS constructs: UAS-myr-RFP, UAS-Lamp-GFP, UAS-N-Cad, UAS-Fas2, UAS-Rst, UAS-*v100*, or UAS-*v100^R755A^*. In addition, we generated a chromosome that contains both UAS-myrRFP and UAS-Lamp-GFP. *v100^R755A^* overexpression and control experiments were done at 18°C, because higher levels of *v100^R755A^* expression in *v100* mutant neurons cause cell death [27].

We used several variations of the MARCM technique [35] to generate positively marked clones with or without the expression of additional reporters or rescue constructs. In these flies, the FRT82B,cl,w+ was replaced with FRT82B,tub-Gal80. The following flies were generated. (1) For *eyFLP^CNS^*: *eyFLP*,*elav*-Gal4;(X*);FRT82B,tub-Gal80/FRT82B,*v100*. (2) For *eyFLP^PRonly^*: *ey3.5FLP*; (Y*); FRT82B,tub-Gal80/FRT82B,*v100*. (X*) stands for one of the following UAS constructs: UAS-Lamp-GFP [37], UAS-pHluorin [58], or UAS-2xFYVE-GFP [36]; (Y*) stands for recombined chromosomes containing GMR-Gal4 and UAS-Lamp-GFP, UAS-pHluorin, or UAS-2xFYVE-GFP.

The following genotype was used to negatively mark clones with RFP: *ey3.5FLP*;GMR-Gal4,UAS-Lamp; FRT82B,UAS-RFP/FRT82B,*v100*.

### Lysotracker Live Imaging

For Lysotracker experiments, brains were removed from third instar lavae and were immobilized on a Sylgard-coated microscope slide using glue stitch. The membrane surrounding the optic lobe was carefully torn so that Lysotracker could enter. Lysotracker Red was added to HL3 at 50 nM. Then 200 µl of this solution was placed onto the prepared tissue, and an image was acquired within 5 min, as recommended by the manufacturer to prevent alkalizing effects. Live imaging was performed as described previously [59].

### Immunohistochemistry, Microscopy, and Image Processing

Dissections were performed as described previously [59]. Brains were fixed in phosphate buffered saline (PBS) with 3.5% formaldehyde for 40–50 min and washed in PBS with 0.4% Triton X-100. High-resolution light microscopy was performed using the a Leica SP5 resonance scanning confocal microscope. Imaging data were processed and quantified using Amira 5.2 (Indeed) and Adobe Photoshop CS4. Fluorescence data were quantified using GraphPad Prism 4. The following antibodies were used at 1∶1,000 dilution: anti-activated Caspase-3, Dlg, Syx7/Avl, Rab7, Sun/CD63, and Syt. Brp (mAb nc82), Chaoptin (mAb 24B10), N-Cad (mAb DNEx8), Rst (mAb 24A5), Flamingo (mAb #74), and Fas2 (mAb 1D4) were used at 1∶50. Guinea pig anti-V100 was used at 1∶2,000. All embryonic immunostaining and assessment of motor axon guidance was done using standard approaches [42] such that whole-mount embryos were fixed, washed in PBS containing 0.1% Triton X-100, and incubated in antibodies to Fas2 (1∶4, 1D4 supernatant, [60]). Brightfield and DIC visualization and imaging were done using a Zeiss Axioimager upright microscope, and images were captured using a Zeiss Axiocam HR camera and Zeiss Axiovision software.

## Supporting Information

Figure S1 **The** ***eyFLP*** **system generates mutant CNS neurons selectively in the visual and olfactory systems.** (A) Whole-mount adult brain. MARCM analysis labeling 50% of all cells affected by *eyFLP* [11] with GFP (arrowheads). Red: Chaoptin immunolabeling of only the photoreceptors (arrows). These are the cells rendered mutant by the *ey3.5FLP* method [28],[29]. (B) P+40% pupal *eyFLP* brain in which heterozygous cells are negatively marked with GFP. Note that the nuclear label Toto-3 (red) is only visible in the absence of GFP (arrowheads). (C) Schematic of the optic lobes in the *Drosophila* brain. Lamina and photoreceptor projections are shown in red, medulla in green, and the lobula complex (composed of lobula and lobula plate) in yellow.
(1.79 MB TIF)

Figure S2 **Loss of** ***v100*** **does not cause apoptosis during development or early adulthood.** Activated Caspase-3 labeling of developing (A and B) and 10-d-old (C) optic lobes. (A and B) Immunolabeling of Caspase-3 (red) in P+15% wild-type (A) and *eyFLP^CNS^ v100* (B) optic lobes reveals no difference in cell death between mutant and control. Green: N-Cad (developing neuropil); blue: Toto-3 (all nuclei). ([A'] and [B'] show Caspase-3 channel only.) (C) Confocal section of the optic lobe cell bodies of a 10-d-old *eyFLP^CNS^ v100* MARCM brain. Mutant cell are marked with GFP; Caspase-3 immunolabeling is in red. ([C'] shows Caspase-3 channel only.) Scale bar in (B) for (A and B): 20 µm. Scale bar in (C): 5 µm.
(3.92 MB TIF)

Figure S3 ***v100^R755A^*** **expression causes heterogeneous guidance receptor accumulations on Syx7-positive membranes and the plasma membrane.** Confocal sections of 1-d-old mosaic eyes in which 50% are mutant for *v100* and express *v100^R755A^* (MARCM), while the other 50% remain wild-type. Approximate clonal boundaries are shown with dotted lines. Immunolabeling for the guidance receptor Dlar is shown in (A), for Rst in (B), and for Fas2 in (C). The boxed regions in (A) and (C) are shown at higher resolution in Figure 5G and 5H. Scale bar in (A) for (A–C): 10 µm.
(4.10 MB TIF)

Figure S4 **Overexpression of guidance receptors in *v100* mutant photoreceptors leads to accumulations that partly colocalize with Syx7-positive compartments.** As shown in Figure 6, at P+30% the guidance receptor Rst exhibits the most prominent accumulations in the developing eye, whereas the guidance receptor Fmi exhibits the most prominent accumulations in photoreceptor terminals. (A) Rst accumulations in the developing eye often partially colocalize with accumulations of the endosomal protein Syx7 (arrows). (B) Accumulations of Fmi in developing photoreceptor terminals also often partially colocalize with Syx7. Scale bar in (A) for (A and B): 5 µm.
(1.74 MB TIF)

Figure S5 **Overexpression of *v100^R755A^* or the guidance receptor Fas2 causes a similar increase of Fas2 accumulations that colocalize with Syx7-positive endosomal accumulations.** Confocal cross-sections of 1-d-old photoreceptor terminals in the lamina are shown. Genotypes are shown on the left. (A) Loss of *v100* leads to heterogeneous accumulations of Fas2 that partially colocalize with the endosomal marker Syx7, albeit rarely. (B) Selective rescue of endosomal sorting with *v100^R755A^* expression in *v100* mutant neurons leads to an increase of Fas2 accumulations that colocalize with Syx7-positive accumulations. (C) Overexpression of Fas2 in *v100* mutant neurons leads to an increase of Fas2 accumulations that colocalize with Syx7-positive accumulations. Scale bar in (A) for (A–C): 5 µm.
(4.66 MB TIF)

Figure S6 **Guidance receptors accumulate in Syx7-positive compartments in the embryonic nervous system.** (A–C) Co-immunolabeling for Fas2 and Syx7 of the ventral ganglion, with cell bodies to the left. Control (*elav-Gal4* only) (A), *v100* null mutant (*v100^4^/Def*) (B), and *elav-Gal4>v100R755A;v100/Def* (C). (D–F) Same as (A–C) except with Robo immunolabeling instead of Fas2. (G) Total Fas2 immunofluorescence; same panel as in Figure 8H. (H) Number of colocalizing pixels for Fas2 and Syx7 for all three genotypes. (I and J) Same as (G and H) but for Robo immunolabeling. In all cases three independent 3-D confocal datasets were quantified. Scale bar in (A) for (A–F): 1 µm.
(4.51 MB TIF)

Figure S7 **Immunolabeling of extracellular DPTP69D reveals no defect in receptor exocytosis.** Confocal sections of embryonic neuromuscular junctions are shown for control (*elav-Gal4*) (A), *v100* mutant (B), and neuronal *v100^R755A^* expression in *v100* mutant embryos (C). (A'–C') Horseradish peroxidase co-labeling to identify neuromuscular junctions. (A″–C″) DPTP69D channel only. The quantification of this data is shown in Figure 8I. Scale Bar in (A) for (A–C): 10 µm.
(1.58 MB TIF)

## Abbreviations

CNS: central nervous system
MARCM: mosaic analysis with a repressible cell marker
P+[number]%: [number]% pupal development
PBS: phosphate buffered saline
