# Supplementary figures and images for: Guidance Receptor Degradation Is Required for Neuronal Connectivity in the Drosophila Nervous System
Source: PLoS Biol. 2010 Dec 7;8(12):e1000553. doi: 10.1371/journal.pbio.1000553 (PMC2998435; doi:10.1371/journal.pbio.1000553)

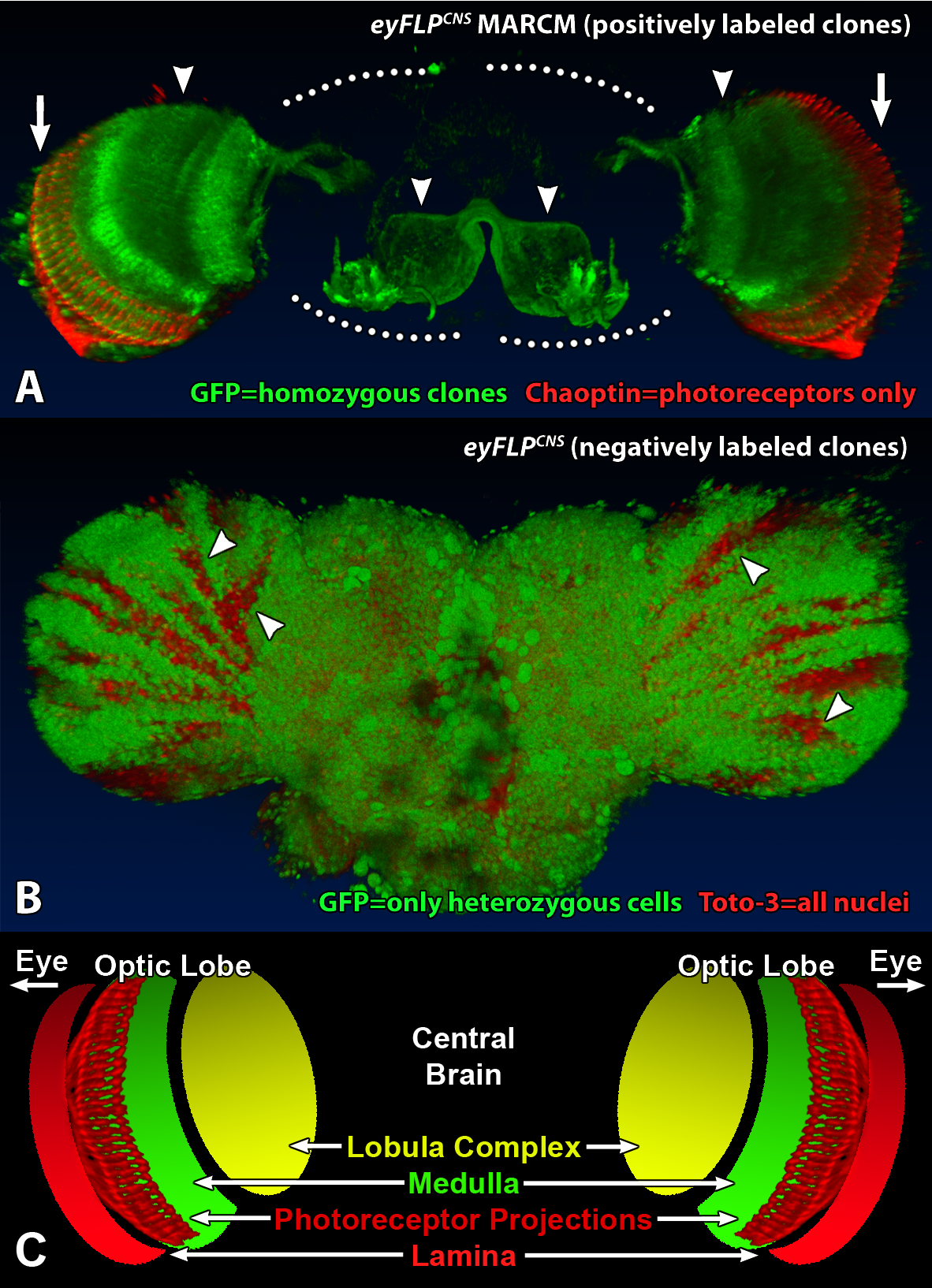

Supplement: Figure S1 — The eyFLP system generates mutant CNS neurons selectively in the visual and olfactory systems. (A) Whole-mount adult brain. MARCM analysis labeling 50% of all cells affected by eyFLP [11] with GFP (arrowheads). Red: Chaoptin immunolabeling of only the photoreceptors (arrows). These are the cells rendered mutant by the ey3.5FLP method [28],[29]. (B) P+40% pupal eyFLP brain in which heterozygous cells are negatively marked with GFP. Note that the nuclear label Toto-3 (red) is only visible in the absence of GFP (arrowheads). (C) Schematic of the optic lobes in the Drosophila brain. Lamina and photoreceptor projections are shown in red, medulla in green, and the lobula complex (composed of lobula and lobula plate) in yellow. (1.79 MB TIF) [file pbio.1000553.s001.tif]

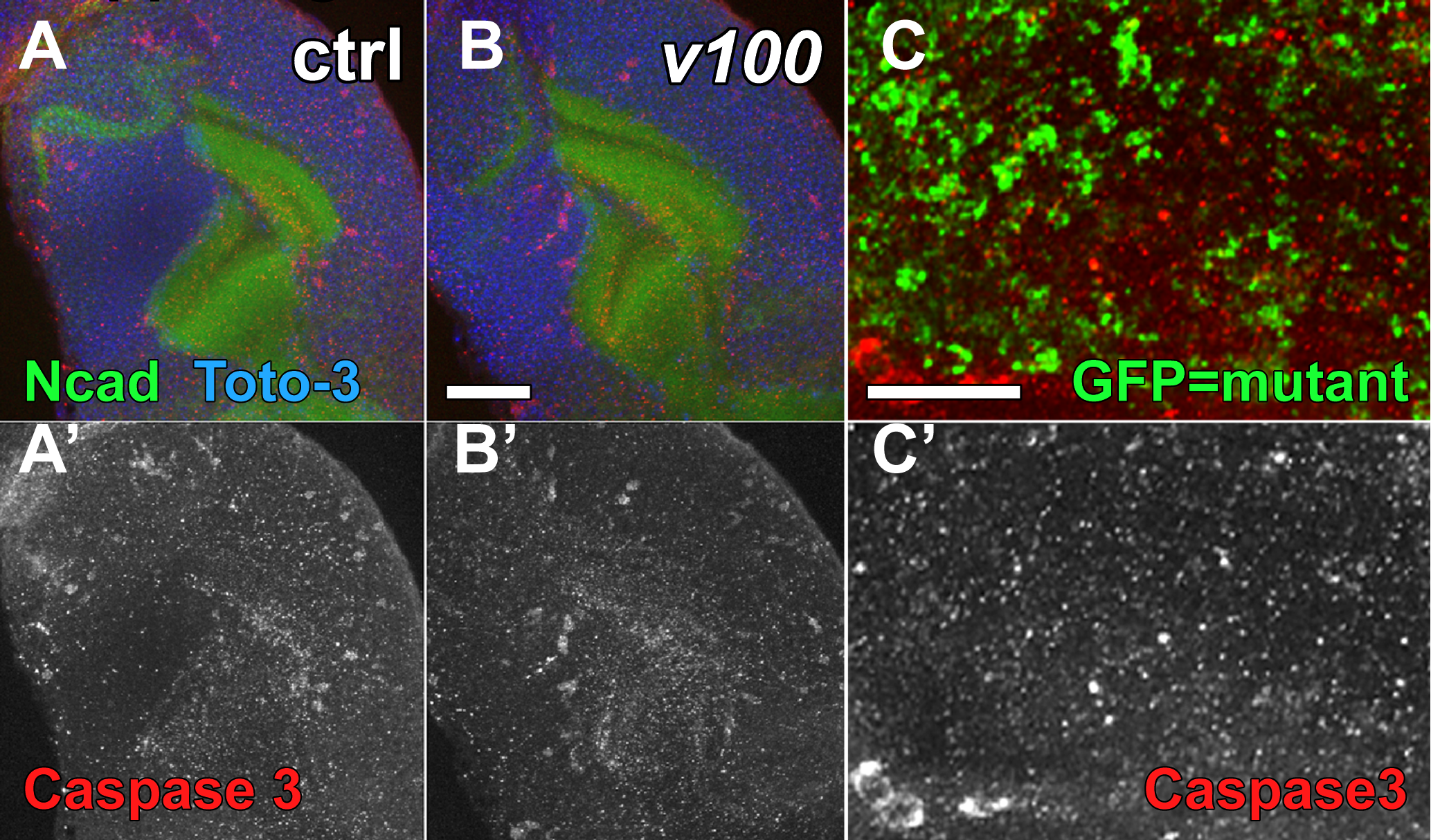

Supplement: Figure S2 — Loss of v100 does not cause apoptosis during development or early adulthood. Activated Caspase-3 labeling of developing (A and B) and 10-d-old (C) optic lobes. (A and B) Immunolabeling of Caspase-3 (red) in P+15% wild-type (A) and eyFLPCNS v100 (B) optic lobes reveals no difference in cell death between mutant and control. Green: N-Cad (developing neuropil); blue: Toto-3 (all nuclei). ([A'] and [B'] show Caspase-3 channel only.) (C) Confocal section of the optic lobe cell bodies of a 10-d-old eyFLPCNS v100 MARCM brain. Mutant cell are marked with GFP; Caspase-3 immunolabeling is in red. ([C'] shows Caspase-3 channel only.) Scale bar in (B) for (A and B): 20 µm. Scale bar in (C): 5 µm. (3.92 MB TIF) [file pbio.1000553.s002.tif]

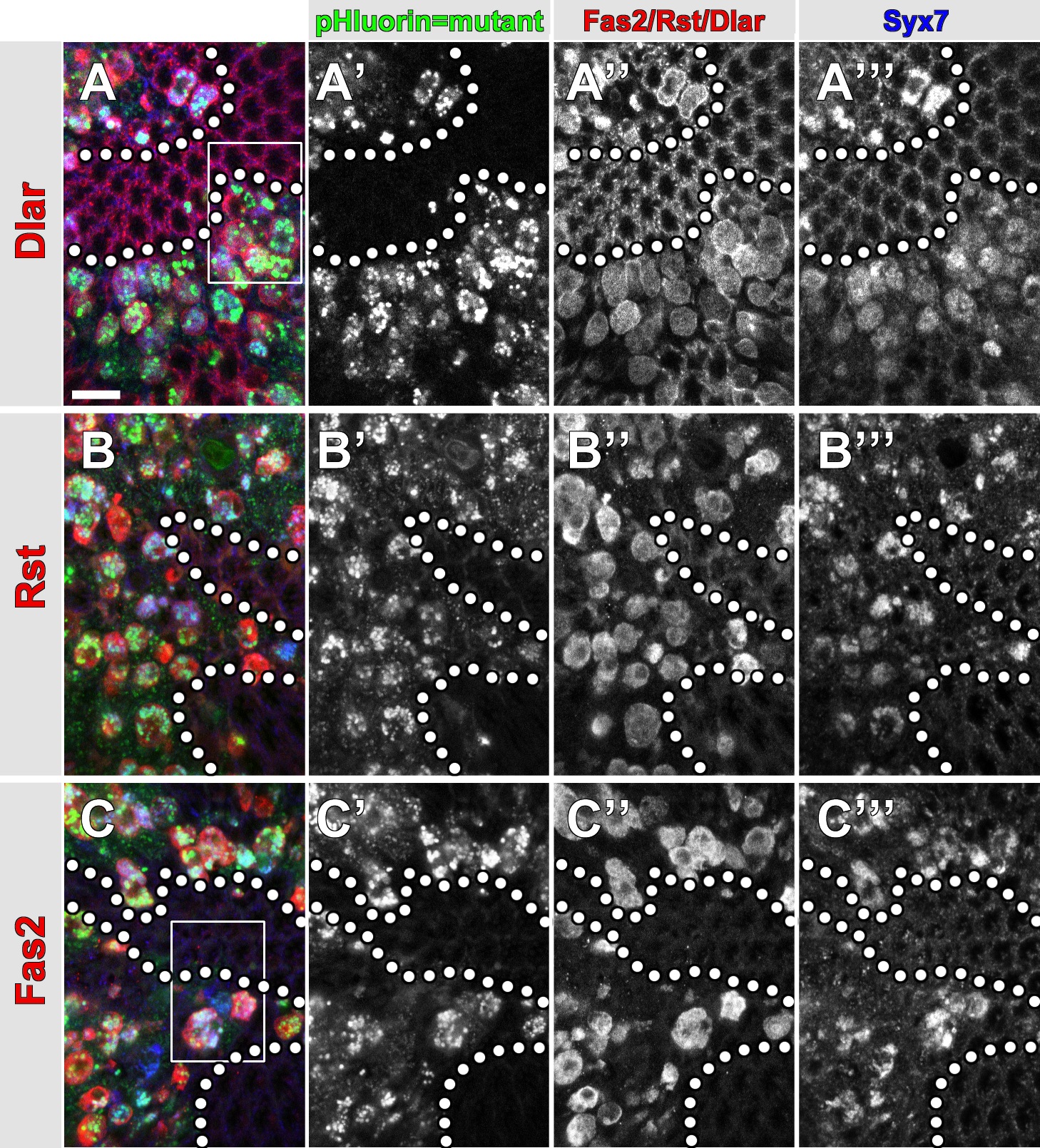

Supplement: Figure S3 — v100R755A expression causes heterogeneous guidance receptor accumulations on Syx7-positive membranes and the plasma membrane. Confocal sections of 1-d-old mosaic eyes in which 50% are mutant for v100 and express v100R755A (MARCM), while the other 50% remain wild-type. Approximate clonal boundaries are shown with dotted lines. Immunolabeling for the guidance receptor Dlar is shown in (A), for Rst in (B), and for Fas2 in (C). The boxed regions in (A) and (C) are shown at higher resolution in Figure 5G and 5H. Scale bar in (A) for (A–C): 10 µm. (4.10 MB TIF) [file pbio.1000553.s003.tif]

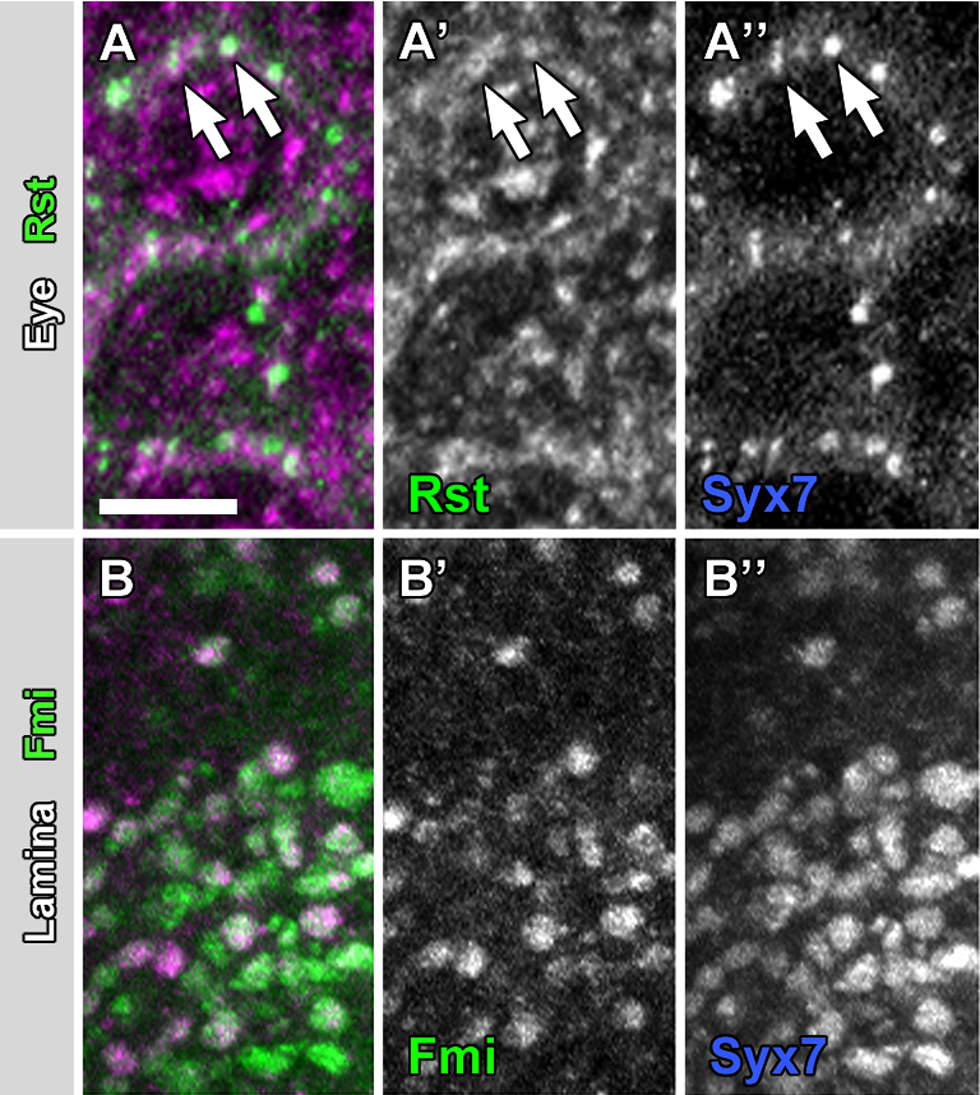

Supplement: Figure S4 — Overexpression of guidance receptors in v100 mutant photoreceptors leads to accumulations that partly colocalize with Syx7-positive compartments. As shown in Figure 6, at P+30% the guidance receptor Rst exhibits the most prominent accumulations in the developing eye, whereas the guidance receptor Fmi exhibits the most prominent accumulations in photoreceptor terminals. (A) Rst accumulations in the developing eye often partially colocalize with accumulations of the endosomal protein Syx7 (arrows). (B) Accumulations of Fmi in developing photoreceptor terminals also often partially colocalize with Syx7. Scale bar in (A) for (A and B): 5 µm. (1.74 MB TIF) [file pbio.1000553.s004.tif]

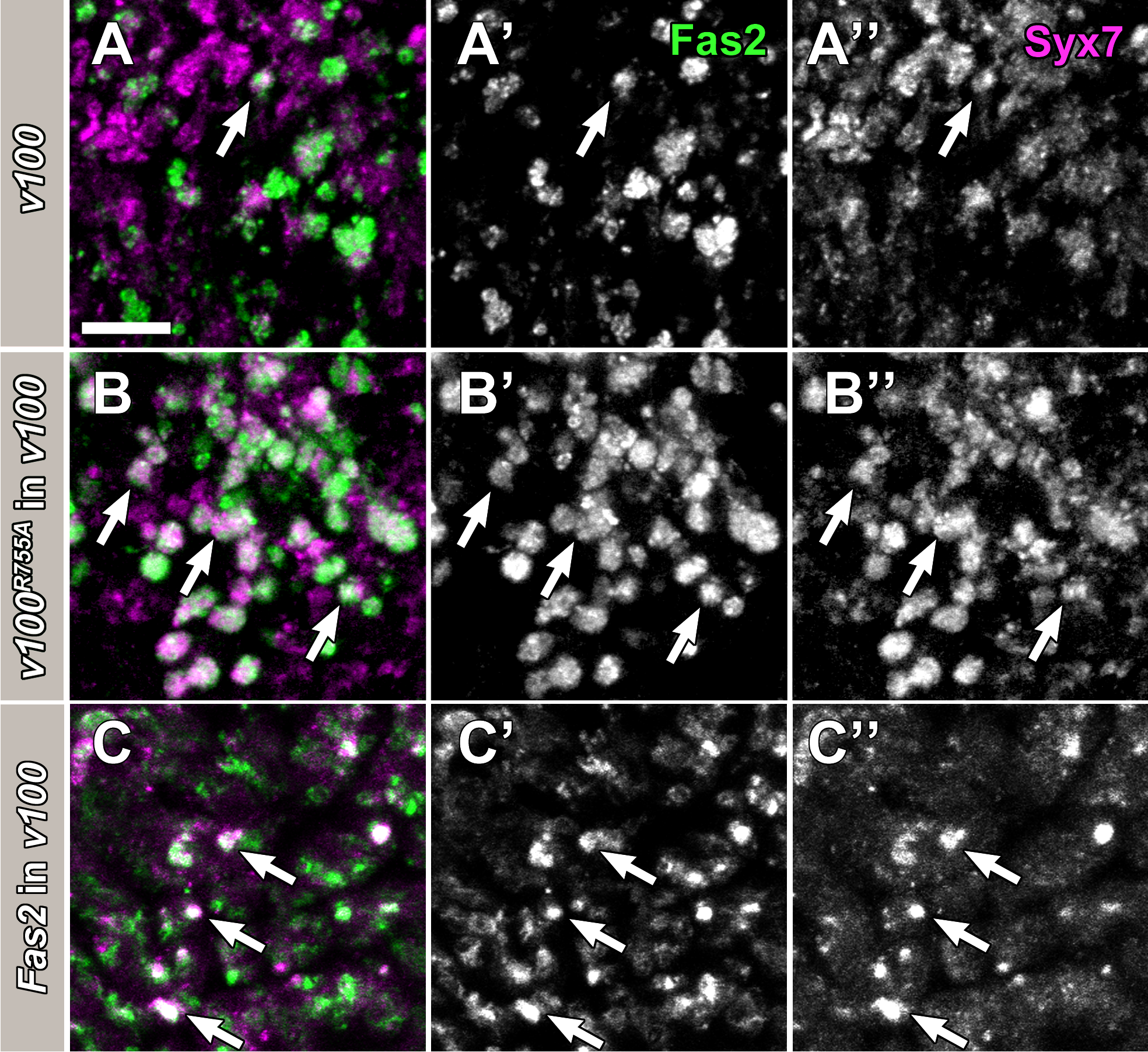

Supplement: Figure S5 — Overexpression of v100R755A or the guidance receptor Fas2 causes a similar increase of Fas2 accumulations that colocalize with Syx7-positive endosomal accumulations. Confocal cross-sections of 1-d-old photoreceptor terminals in the lamina are shown. Genotypes are shown on the left. (A) Loss of v100 leads to heterogeneous accumulations of Fas2 that partially colocalize with the endosomal marker Syx7, albeit rarely. (B) Selective rescue of endosomal sorting with v100R755A expression in v100 mutant neurons leads to an increase of Fas2 accumulations that colocalize with Syx7-positive accumulations. (C) Overexpression of Fas2 in v100 mutant neurons leads to an increase of Fas2 accumulations that colocalize with Syx7-positive accumulations. Scale bar in (A) for (A–C): 5 µm. (4.66 MB TIF) [file pbio.1000553.s005.tif]

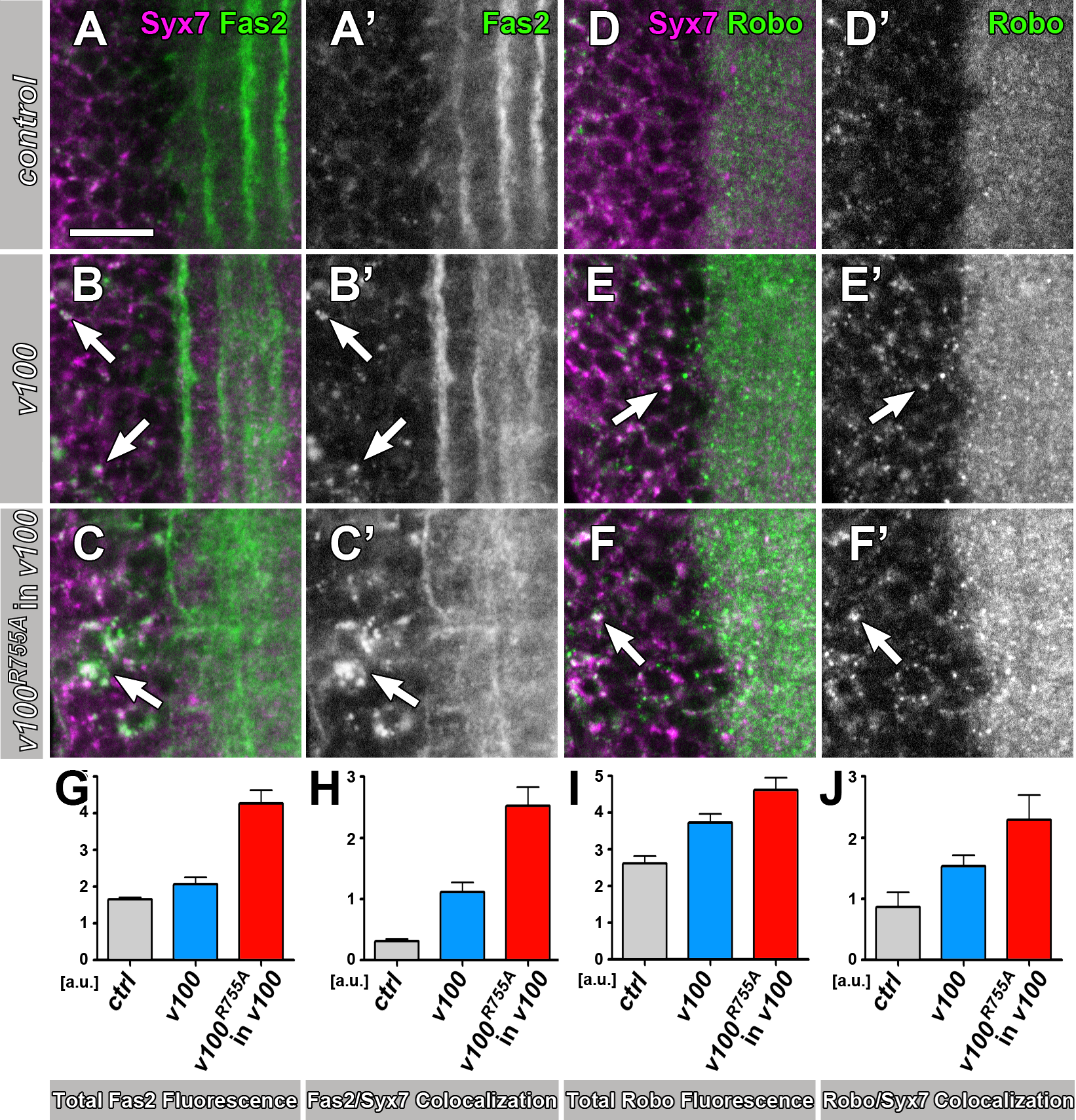

Supplement: Figure S6 — Guidance receptors accumulate in Syx7-positive compartments in the embryonic nervous system. (A–C) Co-immunolabeling for Fas2 and Syx7 of the ventral ganglion, with cell bodies to the left. Control (elav-Gal4 only) (A), v100 null mutant (v1004/Def) (B), and elav-Gal4>v100R755A;v100/Def (C). (D–F) Same as (A–C) except with Robo immunolabeling instead of Fas2. (G) Total Fas2 immunofluorescence; same panel as in Figure 8H. (H) Number of colocalizing pixels for Fas2 and Syx7 for all three genotypes. (I and J) Same as (G and H) but for Robo immunolabeling. In all cases three independent 3-D confocal datasets were quantified. Scale bar in (A) for (A–F): 1 µm. (4.51 MB TIF) [file pbio.1000553.s006.tif]

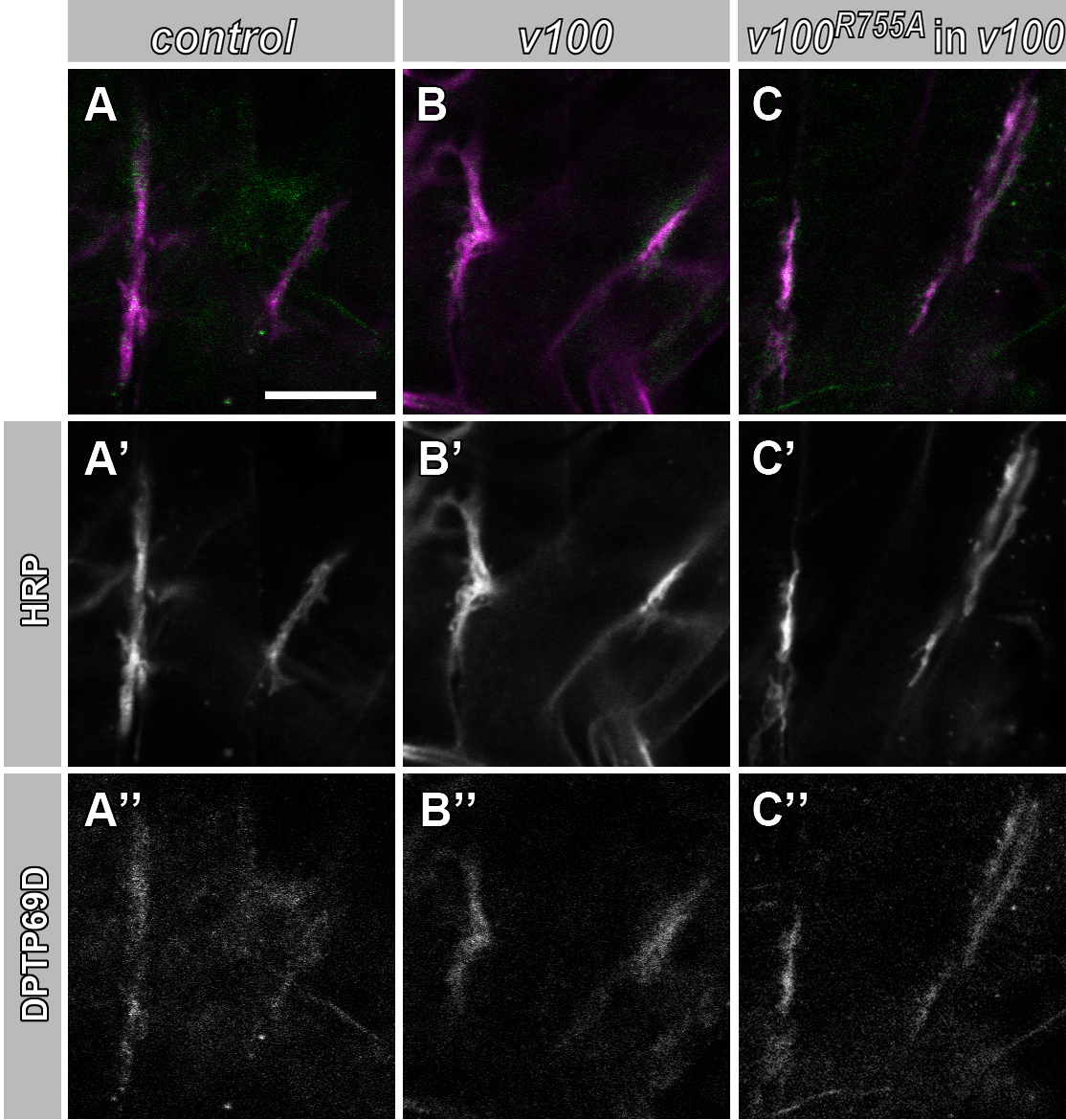

Supplement: Figure S7 — Immunolabeling of extracellular DPTP69D reveals no defect in receptor exocytosis. Confocal sections of embryonic neuromuscular junctions are shown for control (elav-Gal4) (A), v100 mutant (B), and neuronal v100R755A expression in v100 mutant embryos (C). (A'–C') Horseradish peroxidase co-labeling to identify neuromuscular junctions. (A″–C″) DPTP69D channel only. The quantification of this data is shown in Figure 8I. Scale Bar in (A) for (A–C): 10 µm. (1.58 MB TIF) [file pbio.1000553.s007.tif]
